# Pattern of DNA Methylation in *Daphnia*: Evolutionary Perspective

**DOI:** 10.1093/gbe/evy155

**Published:** 2018-07-30

**Authors:** Jouni Kvist, Camila Gonçalves Athanàsio, Omid Shams Solari, James B Brown, John K Colbourne, Michael E Pfrender, Leda Mirbahai

**Affiliations:** 1 School of Biosciences, University of Birmingham, United Kingdom; 2 Department of Statistics, University of California, Berkeley; 3 Centre for Computational Biology (CCB), University of Birmingham, United Kingdom; 4 Department of Biological Sciences and Environmental Change Initiative, University of Notre Dame; 5 Warwick Medical School, University of Warwick, Coventry, United Kingdom

**Keywords:** epigenetics, gene expression, evolution, non-conventional models

## Abstract

DNA methylation is an evolutionary ancient epigenetic modification that is phylogenetically widespread. Comparative studies of the methylome across a diverse range of non-conventional and conventional model organisms is expected to help reveal how the landscape of DNA methylation and its functions have evolved. Here, we explore the DNA methylation profile of two species of the crustacean *Daphnia* using whole genome bisulfite sequencing. We then compare our data with the methylomes of two insects and two mammals to achieve a better understanding of the function of DNA methylation in *Daphnia*. Using RNA-sequencing data for all six species, we investigate the correlation between DNA methylation and gene expression. DNA methylation in *Daphnia* is mainly enriched within the coding regions of genes, with the highest methylation levels observed at exons 2–4. In contrast, vertebrate genomes are globally methylated, and increase towards the highest methylation levels observed at exon 2, and maintained across the rest of the gene body. Although DNA methylation patterns differ among all species, their methylation profiles share a bimodal distribution across the genomes. Genes with low levels of CpG methylation and gene expression are mainly enriched for species specific genes. In contrast, genes associated with high methylated CpG sites are highly transcribed and evolutionary conserved across all species. Finally, the positive correlation between internal exons and gene expression potentially points to an evolutionary conserved mechanism, whereas the negative regulation of gene expression via methylation of promoters and exon 1 is potentially a secondary mechanism that has been evolved in vertebrates.

## Introduction

DNA methylation is an evolutionary ancient epigenetic modification of DNA. It is phylogenetically widespread and is believed to have an integral role in diverse biological processes and species diversity, such as regulation of temporal and spatial gene expression and the development of condition-dependent phenotypic traits (Roberts and Gavery 2012; Sarda et al. 2012). Although DNA methylation mostly occurs at CpG dinucleotide sites across the animal taxa the actual patterns of genomic DNA methylation are highly variable, especially between vertebrates and invertebrates (Feng et al. 2010; Zemach et al. 2010; Keller et al. 2016). DNA methylation pattern can be divided into two main categories, based on distribution and frequency of methylated CpG sites. 1) Vertebrate genomes are globally and heavily methylated at CpG sites, except at putative regulatory regions such as promoters and enhancers. 2) In contrast, invertebrate genomes tend to be sparsely methylated in a mosaic pattern, with the majority of their genomes deprived of methylation, even at promoter regions, which are typically not enriched for CpG dinucleotides compared to the rest of the genome (Feng et al. 2010; Zemach et al. 2010; Jiang et al. 2014; Keller et al. 2016). DNA methylation in invertebrate genomes (Lyko et al. 2010; Xiang et al. 2010; Bonasio et al. 2012; Wang et al. 2013; Song et al. 2017) is predominantly targeted at CpG sites within exons and introns of certain genes (gene bodies). However, it appears that the function of DNA methylation within internal exons is evolutionary conserved. In both vertebrates and invertebrates, gene bodies with substantially enriched DNA methylation are positively correlated with the level of gene transcription (gene expression), suggesting that methylation at these regions has a positive role in gene regulation in both vertebrates and invertebrates. This indicates that the gene body methylation may be an ancient system of gene regulation that evolved prior to when invertebrates and vertebrates diverged, while promoter methylation is a derived system of gene regulation of the vertebrate lineage (Sarda et al. 2012; Keller et al. 2016; Song et al. 2017). However, the evolutionary steps leading to the differentiation of invertebrate and vertebrate genomic DNA methylation remain unresolved.

Evidence that active methyltransferase enzymes and methylated genes are phylogenetically conserved among vertebrates and invertebrates has heightened people’s interest in the diversification of these gene families (Lyko et al. 2010; Xiang et al. 2010; Bonasio et al. 2012; Wang et al. 2013; Song et al. 2017), especially within newly sequenced genomes representing distinct branches of the animal phylogeny. This gene annotation effort has progressed an understanding of the role of DNA methylation in invertebrates, including the first crustacean to have a draft genome assembly, *Daphnia* (Vandegehuchte et al. 2009a, 2009b, 2010a, 2010b; Asselman et al. 2016, 2017). *Daphnia* spp. are fresh-water branchiopods and a recognized model organism by the U.S. National Institutes of Health (Colbourne et al. 2011). It has served as a model organism in various fields of research, including ecotoxicology, ecology, and population genetics for over a century and grows in importance for molecular studies involving neurobiology (McCoole et al. 2012; Toyota et al. 2015; Weiss et al. 2015) and the biology of ageing (Pietrzak et al. 2010; Latta et al. 2011; Dudycha and Hassel 2013; Lohr et al. 2014; Schumpert et al. 2015). Thus, the interest in the methylome of *Daphnia* partly arises from its diverse use as a model organism in a range of research areas, as well as its unique characteristics as a model organism for DNA methylation studies, such as its cyclic parthenogenesis mode of reproduction (Harris et al. 2012). This life history allows the maintenance of large populations of isogenic individuals within the laboratory, providing a unique setup for delineating genetic and epigenetic factors in an experiment. Therefore, *Daphnia* spp. are valuable models for studying the functional effects of DNA methylation in relation to various fields of research. In this study, we provide a comprehensive genome-wide methylation profile for two species of *Daphnia*, which are likely to have diverged for over 200 million years (Colbourne and Hebert 1996). *Daphnia magna* is a member of the subgenus Ctenodaphnia, while *Daphnia pulex* is the nominal species of the subgenus *Daphnia*. Observations are made between different strains and genotypes of *Daphnia* and under intrinsic (ageing) and extrinsic factors; exposure to environmentally relevant concentrations of arsenic, 5-azacytidine (positive control), hyperoxia, and hypoxia. Furthermore, our aim was to achieve an overview of an evolutionary positioning of DNA methylation pattern and function in *Daphnia*. Therefore, we compared the methylome of *Daphnia* with the well-characterized methylomes of two representatives for each of the insecta (*Nasonia vitripennis*, *Apis mellifera*) and mammalia (*Mus musculus* and *Homo sapiens*) classes, representing the two known patterns of DNA methylation in animals (Ball et al. 2009; Lyko et al. 2010; Wang et al. 2013; Ziller et al. 2013; Li and Zhang 2014; Denas et al. 2015; Bewick et al. 2017). This resulted in achieving a better understanding of the potential function of DNA methylation in *Daphnia* spp. across multiple genomic features. Finally, we provide evidence in support of the existence of a set of evolutionarily conserved genes in *Daphnia* that are potentially under DNA methylation regulation.

## Materials and Methods

### *Daphnia* Culturing and Exposure Setup

Cultures of *D.magna* Bham2 strain (originally obtained from the University of Reading, Heckmann et al. 2006) and *D.pulex* Eloise Butler strain (genotypes EB31 and EB45, originally sampled from Eloise Butler pond in Minnesota, Yampolsky et al. 2014) were maintained in HH COMBO and standard COMBO media, respectively as previously described (Kilham et al. 1998; Athanasio et al. 2016). The exposure design followed the OECD guidelines for assessment of chronic toxicity with some modifications (OECD 2012). Briefly, less than 24 h old *D.magna* Bham2 strain were randomly assigned to either control (*n* = 3 replicates, 5 *Daphnia* per replicate) or exposure groups (*n* = 4 replicates, 5 *Daphnia* per replicate). Treatments consisted of 5 days of exposure to 5-azacytidine (3.7 mg L^−1^) with age matched controls (5 days old) or 14 days of exposure to either arsenic (100 µg L^−1^), hypoxia (2 mg L^−1^), or hyperoxia (8 mg L^−1^), all with aged matched controls (14 days old). *Daphnia* in the control groups were maintained under normal laboratory conditions (oxygen concentration: 6 mg L^−1^). Hypoxic and hyperoxic conditions were generated by continuous aeration of the media with 4% and 20% oxygen balanced with nitrogen, respectively. Oxygen concentrations were continuously monitored using an oxygen sensor (Unisense microrespiration system, Denmark). *Daphnia pulex* EB45 and EB31 samples consisted of a pool of 3, 8, and 15 days old *Daphnia* maintained under normal laboratory conditions (*n* = 3 replicates per genotype).

### DNA Extraction and Sequencing

Genomic DNA was extracted from the samples using MasterPure DNA purification kit (Epicentre, USA) following Athanasio et al. (2016). Illumina sequencing library preparation was performed at the Environmental Omics Facility, University of Birmingham, UK. The sequencing libraries were generated using the EpiGenome Methyl-Seq kit (Epicentre, USA), according to the manufacture’s guideline. Non-bisulfite treated *Daphnia* DNA samples (20 ng) as well as bisulfite-treated DNA samples (50 ng) were used for library preparation to calculate the bisulfite conversion efficiency as well as strain and genotype specific variant calling. *Daphnia magna* and *D. pulex* DNA libraries were quantified using KAPA Library Quantification Kit (Illumina), quality checked using TapeStation (Agilent), and sequenced using Illumina HiSeq-2500 platform at the Environmental Omics Facility, University of Birmingham, and Illumina NextSeq 500 platform at the Centre for Genomics and Bioinformatics, Indiana University, respectively. The sequencing run was performed using a rapid run flow cell with paired end and read length of 151 bp for the bisulfite-treated *D. magna* samples (HiSeq), and 80 bp for the non-bisulfite-treated samples and the bisulfite-treated *D. pulex* samples (NextSeq). This project has been deposited at NCBI GEO under accession GSE103939.

### Pre-processing, Mapping, Variant, and Methylation Calling

Illumina adapters (using core sequence: AGATCGGAAGAGC) and nucleotides with low quality (Phred score < 20) were removed with cutadapt (v.1.11; Martin 2011) while processing both read pairs at the same time. The filtered reads [average number of read pairs before and after filtering respectively were 12.73 and 12.68 million for the WGBS (*n* = 28) and 24.14 and 23.27 million for the non-bisulfite converted reference DNA (*n* = 7)] were mapped to the reference genomes of *D.magna* Xinb3 (GCA_001632505.1; Orsini et al. 2016) and *D.pulex* PA42 (GCA_900092285.1; Ye et al. 2017) using BWA Meth (v.0.10; Pedersen et al. 2014) for the bisulfite-treated samples (with an average of 95.35% mapping rate, resulting in 11× coverage) and BWA-MEM (v.0.7.15-r1140; Li and Durbin 2009) for non-bisulfite-treated samples (with an average of 92.53% mapping rate, resulting in 17.9**×** coverage), with default settings. Strain and genotype specific single nucleotide polymorphisms (SNPs) were identified in the non-bisulfite-treated samples with SAMtools mpileup and BCFtools (v.0.1.19; Li 2011). Genome wide SNPs and read depths were identified per sample, with minimum MAPQ score set to 10, without discarding anomalous read pairs as the *Daphnia* genomes are quite fragmented. SNPs that contained indels or had nucleotides other than A/T/C/G in the reference were excluded. SNP calls that had less than 8 reads per sample, or had low confidence (Phred-scaled probability of all samples being homozygous reference < 900) were also removed. In both data sets the filtering resulted in SNP calls that were identical among biological replicates in more than 99% of the cases (99.72% in *D. magna* and 99.97% in *D. pulex*). Furthermore, CpG sites with potential SNPs (with quality score >50) were excluded from the methylation analysis. The genome wide SNP calls and SNPs detected at CpG sites are deposited in NCBI GEO under reference GSE103939.

After removing potential SNP containing CpG sites, methylated CpG sites were called from mapped reads using MethylDackel (v.0.2.1; github.com/dpryan79/MethylDackel). Both uniquely mapped (MAPQ > 10) singletons and discordant reads were retained, while reads with low mapping quality (MAPQ < 10) and nucleotides with low base calling quality (Phred < 30) were excluded. Seven base pairs from both ends of the reads were also excluded, as they showed an excessive amount of methylation potentially due to adapter contamination. The bisulfite conversion rate was calculated from the non-CpG cytosines (∼20 million CHHs) that did not overlap with variable sites identified in the non-bisulfite treated reference samples (*n* = 3 for *D. magna*, *n* = 4 for *D. pulex*). The average bisulfite conversion rate was 99.36% (defined as read count of cytosines converted to thymine/total read count in CHH * 100) (supplementary table S1, Supplementary Material online contains information on read coverage, mapping rate, and bisulfite conversion rate for each sample).

### Differential Methylation Analysis

Differential methylation analysis was performed for destranded CpGs using methylKit (v.1.3.0; Akalin et al. 2012). *Daphnia* spp. have high level of genome duplication (Colbourne et al. 2011) as well as strain specific copy number variation (Keith et al. 2016). Therefore, it is necessary to exclude duplicated regions. This was achieved by excluding CpG sites demostrating coverage greater than 2-fold the standard deviation of the coverage in at least half of the analyzed samples (11/22 for *D. magna* and 3/6 for *D. pulex;* see supplementary fig. S1, Supplementary Material online for an example of excessive coverage) (Scheinin et al. 2014). Furthermore, CpG sites with low coverage (<3 reads) were also excluded from the analysis. To generate a final list of reliable filtered methylated CpG sites for differential methylation analysis, all CpG sites that were saturated (all samples had 100% methylation level), not covered in all samples or had zero or extremely low methylation in most samples (more than half of the samples had <2 methylated reads at the site) were excluded from the analysis. Logistic regression was used to analyze differential methylation between exposure (*n* = 4 replicates per treatment) and control samples (*n* = 3 replicates per corresponding controls). The Q-values were adjusted using the SLIM method (Wang et al. 2011).

### Comparative Methylomics and Pathway Enrichment Analysis

Publicly available data sets of DNA methylation profiles (WGBS) for *H.sapiens*, *M.musculus*, *A.mellifera*, *N.vitripennis*, and *D.magna* Xinb3 were obtained from GEO (Edgar et al. 2002), the ENCODE project (ENCODE Project Consortium 2012), and the Hymenoptera Genome Database (Elsik et al. 2016) (See supplementary table S1, Supplementary Material online for detailed description of the samples, including accession numbers, tissue type, age, gender and treatment group). Methylation levels were calculated for genomic features, 1000 bp upstream from the first exon, each exon and intron, and 1000 bp downstream from the last exon. For genome wide methylation profiling, each feature was scaled to the same relative size, by breaking the features to 101 bins. The methylation level was then averaged across genes for CpG sites with the same relative distance to the start of each feature. CpG sites with zero methylation calls were excluded. CpG densities and clustering were analyzed with CpGcluster, using median distance and *P* value < 1e−5 (Hackenberg et al. 2006). OrthoFinder (Emms and Kelly 2015) was used to identify orthologous gene groups using all available protein sequences (supplementary table S2, Supplementary Material online) for all of the compared species. Orthogroups were assigned to categories (species specific, *Daphnia*/hymenoptera specific, arthropod/mammal specific or common) based on the phylogenetic division in conservation. For the common orthogroups, the methylation levels for each species were calculated by averaging the methylation level of either exons 2–4 (arthoropods), or exon 1 (mammals) for each gene and then taking the mean across all genes in the same orthogroup. Furthermore, to conduct pathway enrichment analysis using Reactome pathways (Fabregat et al. 2016), methylated CpG sites were analyzed at the gene level with ClusterProfiler (Yu et al. 2012). As most arthropod species are not annotated in Reactome, protein blast was used (with e-value < 1e−20) to find orthologous genes in humans. The reference genes (universe) for the enrichment analysis were limited to only those human genes that were identified by blast.

In addition to species level comparison of DNA methylation and gene expression, a separate more detailed analysis of the DNA methylation similarities and differences among the *Daphnia* “control” samples was conducted. Instead of using orthogroup averages in methylation, homologous genes (10,101 genes) identified with direct reciprocal blastp (with e-value < 1e−20) were used. The genes were ranked based on the maximum methylation levels of CpGs located within unique exons. CpGs that overlapped with multiple genes (including 1 kb upstream and downstream regions) were excluded as well as CpGs that were not covered in all samples, with at least 3 reads. The genes were clustered based on the ranked methylation levels. In addition, sub-clusters, identified with cutree (R Core Team 2018), that showed the most or least similarities between the two species were analyzed for pathway enrichment using Reactome as described above.

### Phylogeny of DNA Methyltransferases

The phylogenetic analysis was done for all six species (*H.sapiens*, *M.musculus*, *A.mellifera*, *N.vitripennis*, and *D.magna* Xinb3 and *D.pulex* PA42) with all of the protein sequences identified as orthologous to the human DNA methyltransferase (DNMT) genes in the OrthoFinder analysis (supplementary table S2, Supplementary Material online). A maximum likelihood phylogeny was constructed for the DNMT-proteins using the Phylogeny.fr pipeline with the default settings (Dereeper et al. 2008), without using the conserved domain selection with Gblocks (v.0.91b; Talavera et al. 2007). Briefly, the sequences for each DNMT gene (supplementary file S1, Supplementary Material online) were aligned using MUSCLE (v.3.8.31; R. C. Edgar 2004), the phylogenies were constructed using PhyML (v.3.1; Guindon et al. 2010) and the trees were rendered with TreeDyn (v.198.3; Chevenet et al. 2006) and modified in TreeGraph (v.2.14.0; Stöver and Müller 2010). The *Arabidopsis thaliana* MET1 gene was used as an outgroup.

### Gene Expression Analysis of *D. pulex* Eloise Butler and *D. magna* Bham2

RNA was extracted from the same *D. pulex* and *D. magna* (5 days old) samples used in the WGBS analysis using RNeasy micro kit (Qiagen) according to the manufactures protocol. Sequencing libraries were prepared using the Illumina TruSeq stranded mRNA kit and sequenced using Illumina NextSeq 500 and HiSeq-2500 platforms at the Centre for Genomics and Bioinformatics, Indiana University and Environmental Omics Sequencing Facility, University of Birmingham, UK, for *D. pulex* and *D. magna*, respectively. Single end 85 bp RNA-seq reads for *D. pulex* and paired end 150 bp RNA-seq reads for *D. magna* were filtered with cutadapt (v.1.11; Martin 2011) the same way as the WGBS reads and mapped to the reference genomes of *D.pulex* PA42 (GCA_900092285.1; Ye et al. 2017) and *D.magna* Xinb3 (GCA_001632505.1; Orsini et al. 2016) using TopHat2 (v.2.1.0; D. Kim et al. 2013). The read counts for genes were extracted with HTSeq (v.0.9.1; Anders et al. 2015) using unstranded union mode for genes. Gene expression in *D. magna* Bham2 samples were compared to age matched control samples in *D. magna* Xinb3 obtained from an independent study (Orsini et al. 2016), using DESeq2 (Love et al. 2014). As the gene expression estimates for Xinb3 are based on transcriptome models, RNA-seq reads mapping to overlapping genes are potentially counted more than once, whereas genome mapping and read counting with HTSeq excludes this category of reads entirely. For this reason we removed overlapping genes (7,033/21,293 genes with overlapping exons) from the gene expression comparisons. The read counts were converted to FPKM values for gene expression and DNA methylation comparisons (Trapnell et al. 2010). The data generated for this study have been deposited to NCBI GEO under reference GSE103939.

### Correlation of Gene Expression and DNA Methylation Data

Publicly available RNA-seq data, matching to the WGBS data, for *H.sapiens*, *M.musculus*, *A.mellifera*, *N.vitripennis*, and *D.magna* Xinb3 were obtained from GEO (Edgar et al. 2002), the ENCODE project (ENCODE Project Consortium 2012), and the Hymenoptera Genome Database (Elsik et al. 2016) (supplementary table S1, Supplementary Material online). To analyze the correlation between DNA methylation and expression level, the mean methylation level for each gene was calculated for exon 1, exons 2–4, and 1 kb upstream from the first exon. The CpG sites with zero methylation were excluded as they dominated most of the arthropod species. The genes with similar methylation levels were grouped together into methylation quantiles and the average methylation level of the group was regressed against the average expression (FPKM) level of those genes.

### Conservation of Methylation and Gene Expression Levels

To investigate if genes with high levels of DNA methylation and gene expression were more likely to be evolutionarily conversed compared to the genes with low methylation and low expression levels, we compared the methylation and expression densities between the different categories assigned as species specific, *Daphnia*/hymenoptera specific, arthropod/mammal specific or common by the OrthoFinder program. A joint clustering of DNA methylation and gene expression was carried out for the genes that were common across all species, as identified in the OrthoFinder analysis. Mean expression and mean methylation values were calculated for each gene and then averaged for the whole orthogroup. For the arthropod species, methylation levels were based on exons 2–4 and for vertebrates based on exon 1, as these were most strongly correlated with gene expression levels. The methylation and expression levels were then scaled from 0 to 1. For vertebrates the scale was reversed as the methylation level of exon 1, was negatively correlated with gene expression.

## Results

### DNA Methylation is Conserved and Follows a Bimodal Distribution Across Vast Taxonomic Distances

Eukaryotic DNMTs are key enzymes that methylate DNA and are remarkably conserved in structure and function across different species. While DNMT1 is responsible for maintenance of DNA methylation during DNA replication, DNMT3 family of enzymes are responsible for *de novo* establishment of DNA methylation (Zhong 2016). Therefore, to conduct phylogenetic analysis of DNMTs, all available protein sequences including DNMTs were retrieved from NCBI for *H.sapiens* (human, GCA_000001405.15), *M.musculus* (mouse, GCA_000001635.7), *D.magna* (GCA_001632505.1), *D.pulex* (GCA_900092285.1) and from hymenopteragenome.org for *A.mellifera* (honey bee, Amel_4.5) and *N.vitripennis* (wasp, Nvit_1.2)*.* The human DNMTs were used to find and confirm the orthologous sequences in the other species, including *D.magna* (Xinb3) and *D. pulex* (PA42), using OrthoFinder. A maximum likelihood phylogeny (supplementary fig. S2, Supplementary Material online) was constructed for all of the protein sequences identified (supplementary file S1, Supplementary Material online). In all of the species studied all three DNMT genes (DNMT1, TRDMT2, and DNMT3) could be identified. All of the protein sequences clustered together in the correct DNMT gene families. Within the gene families the proteins clustered by species except for one protein in both *Daphnia* species. In *D. magna*, one DNMT1 protein (Dapma7bEVm024669t1) clustered together with mouse DNMT1 proteins. This protein however is only identified by *de novo* transcriptome sequencing and has no matching sequence in the genome assembly. In *D. pulex*, one DNMT1 protein (gene10115) clustered apart from all other DNMT1 proteins. This gene however had no read support in the re-sequenced reference samples (*n* = 4 with average 11× median coverage).

**Fig. 1.**
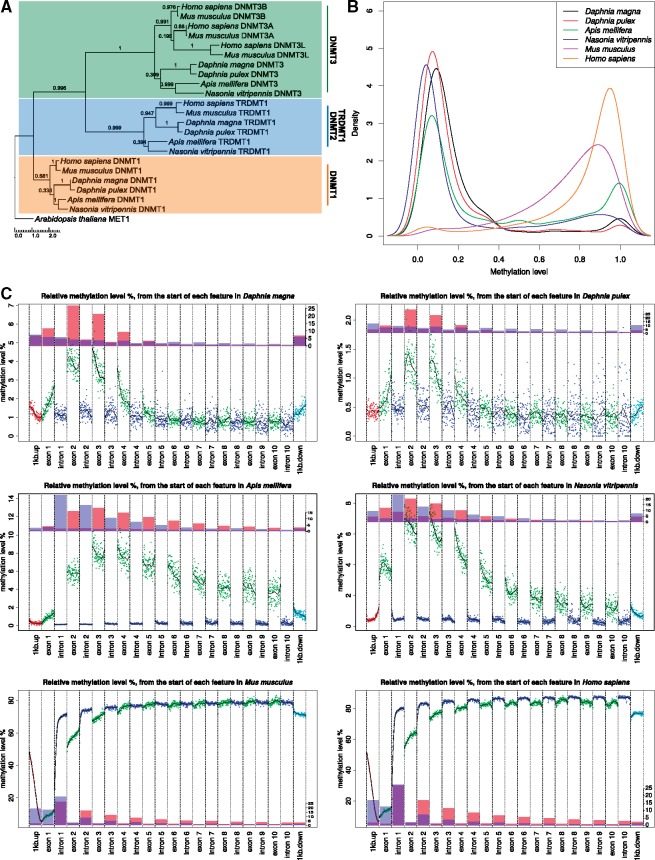
—Genomic overview of DNA methylation levels in four arthropods and two vertebrates. (*A*) Maximum likelihood phylogeny of methyltransferase proteins in the study species. The phylogeny was estimated for all methyltransferase proteins identified in OrthoFinder analysis, using *A. thaliana* MET1 as an outgroup. One representative protein for each methyltransferase gene was selected for each species for visualization. The numbers above the branches are aLRT (approximate likelihood ratio test) support values in the phylogeny. The scale bar shows the expected number of amino acid substitutions. (*B*) A density plot of global methylation levels among the species. CpG sites with zero methylation were excluded from the analysis. (*C*) Methylation landscape across genomic features. The average methylation level was calculated for CpGs with similar relative distance (0–100) from the start of each genomic feature (exons, introns, 1 kb upstream and downstream of the first and last exon). A loess fit was calculated across each feature. The bar plots represent the relative abundance of high methylated CpGs (methylation level > 50%, HM = red) and LM CpGs (methylation level < 50%, LM = blue) across the genomic features.

A reduced phylogeny with a single (highest blastp score to human DNMT genes) representative for each DNMT gene is shown in figure 1*A*. The amino acid conservation for the entire gene length was fairly low (average 37.57%), while for the conserved domains (Gblocks) the conservation was much higher (average 58.48%). In all arthropods the DNMT1 gene contained five superfamily domains (DNMT1-RFD, zf-CXXC, BAH, Dcm, AdoMet_Mtases), TRDMT1 contained two domains (AdoMet_MTases, Dcm), and DNMT3 gene contained three domains in all species (FYVE_like_SF, Dcm, AdoMet_Mtases). In DNMT3 the PWWP-domain was missing in both *Daphnia* species, but present in *A.mellifera* and *N.vitripennis*, and the PHD_SF-domain was missing only in *D.magna* (supplementary table S3, Supplementary Material online).

To profile the *Daphnia* species methylome and to achieve a better understanding of the level of variation in the methylome of *Daphnia* species, we performed whole genome bisulfite sequencing (WGBSeq) of adult *D.magna* Bham2 strain and *D.pulex* Eloise Butler strain (genotypes EB31 and EB45). In addition, we used a WGBSeq data set for the inbred *D.magna* Xinb3 strain downloaded from GEO (GSE60475; Asselman et al. 2016). In order to compare the DNA methylation pattern of *Daphnia* against other invertebrate and vertebrate species, we used WGBSeq data from *H.sapiens*, *M.musculus* (ENCODE Project Consortium 2012), *A.mellifera* (Lyko et al. 2010), and *N.vitripennis* (Wang et al. 2013) downloaded from GEO and the ENCODE project (supplementary table S1, Supplementary Material online)*.* As demonstrated in supplementary table S4, Supplementary Material online, the CpGs are more heavily clustered in the vertebrate species (∼14% of CpGs are in CpG clusters, with an average distance of 112 bp), compared to the arthropod genomes (∼2% CpGs in clusters, average distance of 25 bp). Although overall the distribution of CpG clusters in *Daphnia* species are more similar to wasps than the over two mammal species, there are some differences between *Daphnia* species and wasps. In *D. magna* and *D. pulex* there are 2,937 and 6,393 CpG clusters with an average length of 94 bp and 88 bp and 16 and 15 CpGs per cluster, respectively. These clusters contain only 1.07% and 1.7% of all CpGs in the genomes of *D. magna* and *D. pulex* respectively. However, *A.mellifera* and *N.vitripennis* have 2.7–6.3 times more CpG clusters compared to the *Daphnia* species, although the percentage of CpGs within the clusters is approximately the same (1.9–2.9%). More interestingly, the CpG clusters in *Daphnia* species are enriched in exonic regions (*Daphnia*: 35.6% vs. wasp: 6.6%) while in wasps the CpG clusters are mainly enriched in intronic regions (*Daphnia*: 13.1% vs. wasp: 46.6%).

In all species, the methylation levels of CpG sites can be divided to two clear categories of high methylation levels (HM: methylation level above 50%) and low methylation levels (LM: methylation level below 50%). For *H.sapiens* and *M.musculus*, majority of the CpG sites are highly methylated (84.90% and 79.29%, respectively) while for *A.mellifera*, *N.vitripennis*, *D.magna*, and *D.pulex* majority of the CpG sites show low levels of methylation (0.60%, 0.67%, 0.74%, and 0.19%, respectively). This results in extremely low levels of global DNA methylation in invertebrates (0.4–1.5%) compared to vertebrates (72–76%). However, in all species high methylated CpGs are under-represented in CpG clusters (supplementary table S5, Supplementary Material online) and the frequency of methylation across all species follows a bimodal distribution (fig. 1*B*), including *Daphnia* (Dip-test D = 0.0053182, *P* value < 2.2e−16). As shown in supplementary table S6, Supplementary Material online, HM sites are mainly located at intron regions (∼87.15%) in the two mammalian species while in the arthropods they are mainly located at exon regions (∼76.95%), specifically in exons 2–4 (fig. 1*C*). For example, in *D. magna* 73.5% of the HM are located within exons (chi-squared = 4,350.2, *P* value < 2.2e−16) while 5ʹ-UTR regions, 3ʹ-UTR regions, and introns only contain on average 6.6%, 13.3%, and 6.6% of the HM, respectively. This pattern is similar in *D. pulex, A. mellifera*, and *N. vitripennis.* In addition, overall methylation pattern is different between vertebrates and invertebrates (fig. 1*C*). In vertebrates, the methylation level gradually increases until exon 2 and then remains high throughout the remaining introns and exons while in the investigated invertebrate species methylation levels decreases after the first four exons towards the 3ʹ region (fig. 1*C*).

### DNA Methylation Level and Species Conservation and Divergence

**Fig. 2.**
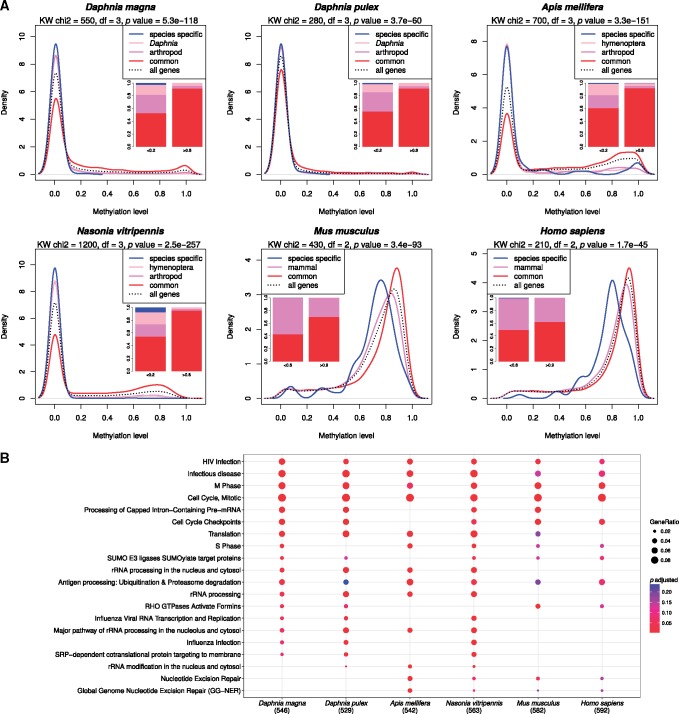
—Conservation of high methylated genes. (*A*) Density plot of methylation averages of genes separated into different evolutionary conservation categories identified by OrthoFinder from least conserved to most conserved (species specific, *Daphnia*/hymenoptera, arthropod/mammal, and common). The bar plots show the scaled proportion of each category of genes at selected methylation ranges. (*B*) Enrichment analysis of genes with the highest methylation levels, independently selected in each species. Genes were ranked by their methylation level and the top 1,000 highest methylated genes were selected. The annotations are based on *H. sapiens* orthologs identified with blastp (best match, with e-value < 1e−20).

To investigate if there is a link between DNA methylation levels and evolutionarily conservation of genes, we separated the genes in our six species into distinct categories (species specific, *Daphnia*/hymenoptera specific, arthropod/mammal specific or common), by identifying orthologous gene groups with OrthoFinder. The genes with high levels of DNA methylation were significantly enriched for the common category (evolutionarily conserved), while species specific genes had much lower methylation level (fig. 2*A*). As shown in figure 2*A*, there is a decrease in the density of common > arthropod > *Daphnia* specific and > species specific genes moving from high to low methylation level (*x* axis). We selected the top 1,000 genes with highest methylation levels per species (based on the average methylation levels in exons 2–4) and conducted a pathway enrichment analysis using Reactome. This analysis showed that high methylated genes are mostly enriched for the same pathways across species (fig. 2*B*). Many of the pathways that were shared were enriched for RNA-processing pathways, cell cycle regulation and processes that respond to viral infections (such as HIV) (fig. 2*B*, supplementary table S7, Supplementary Material online).

**Fig. 3.**
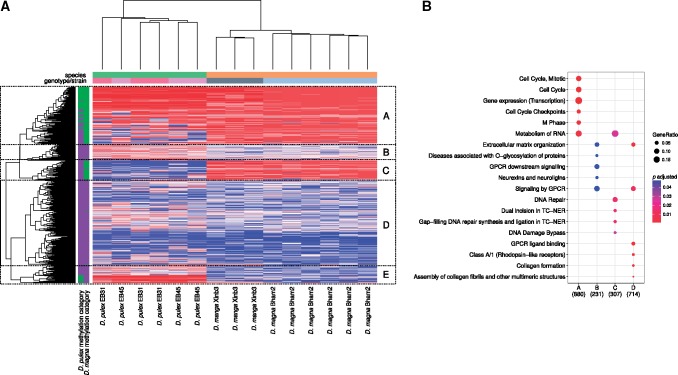
—Differences and similarities in methylation in homologous genes of *D. pulex* and *D. magna*. (*A*) Heatmap of ranked methylation levels (red = high, blue = low) of homologous genes in control samples, identified with reciprocal blastp (with e-value < 1e−20). Genes were ranked by the maximum methylation levels of CpGs located within unique exons, and the sub-cluster (*A*–*E*) were identified with cutree. The side panel shows the average methylation level in two categories (green >50%, purple <50%) for both species. The top panel shows the species, strain, and genotypes in different colors that correspond with the sample names at the bottom. (*B*) Enrichment analysis of genes within sub-clusters, using Reactome, shows the top five significantly enriched categories for each cluster (see supplementary table S8, Supplementary Material online for a comprehensive list).

**Fig. 4.**
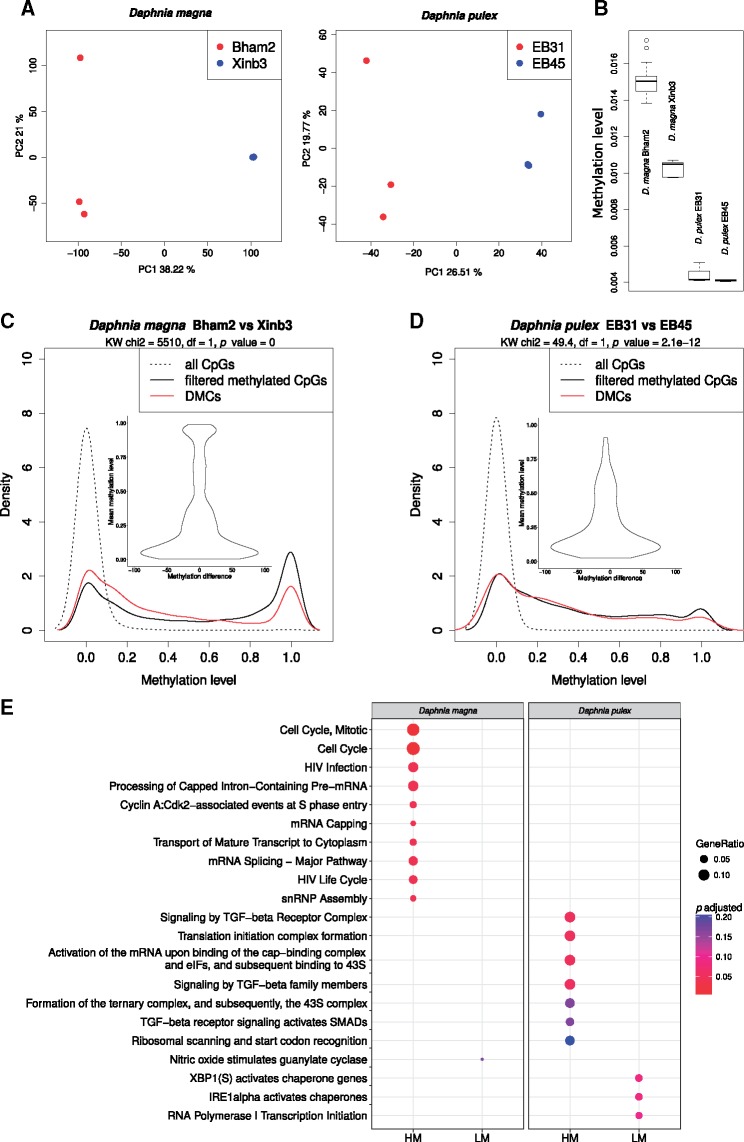
—Differential methylation between strains of *D. magna* (Bham2 vs. Xinb3) and genotypes of *D. pulex* (EB31 vs. EB45). (*A*) Principal component analysis (PCA) of methylation levels in *D. magna* strains Xinb3 and Bham2, and in *D. pulex* genotypes EB31 and EB34, using a filtered data set; CpGs with low coverage (<3 reads in each sample) or extremely low methylation levels (<2 methylated reads in at least half of the samples) were excluded. Only age matched control samples were used in each comparison (*n* = 6). (*B*) Boxplot of global methylation level in *D. magna* (Bham2 and Xinb3 strains) and *D. pulex* (EB31 and EB45 genotypes). (*C*) Density plot of methylation levels of the DMCs (red), contrasted to both unfiltered CpGs (dashed line) as well as filtered CpGs (solid black). The violin plots within the density plots show the magnitude of difference relative to the methylation level in the DMC sites. (*E*) Enrichment analysis of DMCs among *D. magna* strains (Bham2 vs. Xinb3) and *D. pulex* genotypes (EB31 vs. EB45). The analysis was performed separately for the genes containing high methylated CpGs (HM) and genes containing exclusively LM CpGs in the two different species.

Although the methylation pattern across genomic features is the same between genetically diverse *Daphnia* species, strains and genotypes (fig. 1*C*), there are gene specific methylation differences between the species, strains and genotypes, indicating a possible link between gene specific DNA methylation difference and genetic diversity within a genus (figs. 3 and 4*A*). Furthermore, global methylation levels varied between the two *D. magna* strains from 1.51% to 1.03% (in Bham2 and Xinb3, respectively), and between the two *D. pulex* genotypes from 0.44% to 0.41% in *D.pulex* EB31 and EB45, respectively (fig. 4*B*). Therefore to further investigate the link between genetic diversity and DNA methylation within a genus, we conducted a more detailed analysis of DNA methylation differences between different species (*D. pulex and D. magna*), different strains (*D. magna* Xinb3 and Bham2) and genotypes (*D. pulex* genotypes EB31 and EB45) of *Daphnia*. The different strains and genotypes of *Daphnia* are genetically quite diverse as evident by the amount of SNP variation observed among them. The *D. magna* Bham 2 strain has 239,174 fixed (homozygous) SNPs compared to the Xinb3 strain (reference genome) and the *D. pulex* EB strain has a similar number of fixed SNPs (286,828) compared to the PA42 strain (reference genome). On the other hand, the two genotypes of the *D. pulex* EB strain (EB31 and EB45) are a lot more similar to each other, containing only 60,984 fixed SNPs (21.26% of all fixed SNPs) between them. Approximately 10% of the fixed SNPs overlap with CpGs in the genomes in both species (9.24% in *D. magna* Bham2 vs. Xinb3, 10.10% in *D. pulex* EB vs. PA42, and 12.90% *D. pulex* EB31 vs. EB45), which is a significant enrichment considering the overall occurrence of CpGs within the genome (8.24% on average). The enrichment is higher in *D. pulex*, which has more CpGs compared to *D. magna* (*D. pulex* EB vs. PA42: chi-squared = 1,086.8, *P* value < 2.2e−16, *D. pulex* EB31 vs. EB45: chi-squared = 1,413.4, *P* value < 2.2e−16 and *D. magna* Bham vs. Xinb3: chi-squared = 210.19, *P* value < 2.2e−16).

We identified 10,101 homologous genes between the two *Daphnia* species. From this list, approximately half of the genes were excluded from the analysis as they either overlapped with other genes or did not have sufficient read coverage in all samples. This resulted in confidant clustering of 5,302 homologous genes in the two *Daphnia* species by ranked methylation levels. As shown in the heatmap (fig. 3*A*), the two species are quite distinct in regards to methylation levels of homologous genes, having multiple clusters where the methylation level is high (>50%) in only one species and low (<50%) in the other (Clusters C and E in fig. 3*A*). In contrast, there are much fewer differences between the strains and genotypes of each species. In fact, the clustering fails to resolve the two genotypes of *D. pulex* from each other. The genes in cluster B (with 8.4% of the genes) that demonstrate higher methylation level in *D. pulex* compared to *D. magna* are enriched for O-glycosylation of proteins, extracellular matrix organization, and multiple signaling pathways (GPCR, neurexin, and neuroligins). The other cluster (E, with 9.4% of genes) that contained genes with very high methylation (>50%) in *D. pulex* and low methylation (<50%) in *D. magna* is not significantly enriched for any pathways, but still contains many genes in the same signaling pathways as identified in cluster B. While the genes that have high methylation (>50%) in *D. magna* and low methylation (<50%) in *D. pulex* (cluster C, with 10.3% of genes) are enriched for DNA repair and RNA metabolism. Genes that have high methylation in both species (cluster A, with 29% of genes) are enriched for the same pathways as identified before (Cell cycle, infections and gene expression). Genes with low methylation in both species (cluster D, with 42.8% of genes) are enriched for extracellular matrix organization, signaling (GPCR) and collagen processing (fig. 3*B* and supplementary table S8, Supplementary Material online).

### Methylation Divergence Between Subspecies (Strains and Genotypes of *Daphnia*)

We analyzed both species separately for differential methylation in individual CpGs by comparing the age matched control samples of the two strains of *D. magna* against each other, and the two genotypes of *D. pulex* against each other. There are 20,656 differentially methylated CpGs (DMC, FDR < 0.05) between *D.magna* strains, Bham2, and Xinb3 (fig. 4*C*, supplementary table S9, Supplementary Material online).

Even though at a global level there is only a 0.5% difference in methylation level between *D. magna* Bham2 and *D. magna* Xinb3, almost all of the CpGs (>90%) have zero methylation in both strains. To compare the magnitude of the methylation level of the CpG sites that are methylated (methylation level above zero) between the two *D. magna* strains, we filtered out the CpGs that are consistently unmethylated or not covered in all samples (filtered methylated CpGs). This showed that the difference in methylation levels of the filtered methylated CpGs is 10× fold (5.01%) higher compared to the global average (0.5%) between the two strains. The higher global DNA methylation level observed for *D. magna* Bham2 is also retained at the level of DMCs. Majority of the DMCs (75%) have higher methylation in Bham2 (8.02% higher on average), which is still a significant enrichment compared to the filtered methylated CpGs (chi-squared = 243.78, *P* value = 5.90e−55). Overall, more than 73% of the DMCs belong to the category of LM (∼1/3 of DMCs have methylation levels below 10%; fig. 4*C*). Furthermore, LM demonstrated a greater magnitude of change in methylation level compared to HM between the two strains (fig. 4*C*, violin plot). Even though most DMCs are low methylated (LM) in both strains, there are a few DMCs (4.30%) where the methylation switches from near zero to near 100% (methylation difference > 90%). These DMCs belong to a wide range of genes such as DNA damage repair proteins (like RAD51B) and heat shock proteins (hsc70 interacting protein), transcription (Sp3) and splicing factors (U2AF 65K), proteases (Proteasome subunit alpha type-1), signaling molecules (Neurexin IV), and structural components (Tubulin). A complete list of DMCs between the two strains and their methylation levels are presented in supplementary table S9, Supplementary Material online).

As shown in supplementary figure S3A, Supplementary Material online the genes categorized as common (evolutionarily conserved) are slightly under-represented in the differentially methylated genes between the two strains of *D. magna*. In contrast to *D. magna*, the two *D. pulex* genotypes have very few methylation differences (differentially methylated CpGs: 1,442, FDR < 0.05). However, similar to *D. magna* more than 80% of the differentially methylated CpGs belong to the category of LM and are also located within gene bodies (78%) and mostly within exons (63.25%; fig. 4*D*). As shown in supplementary figure S3B, Supplementary Material online, there is a significant enrichment for the genes categorized as common (evolutionarily conserved) in the differentially methylated genes between the two genotypes of *D. pulex*. Similar to *D. magna* the slight difference in global methylation levels between the two genotypes, EB31 and EB45, is also observed in the DMCs, with EB31 having higher methylation in 58.18% of the DMCs (chi-squared = 18.903, *P* value = 1.375e−05). There are only 11 DMCs with methylation difference above 90% between the two genotypes and these DMCs are detected in the following genes: dual specificity protein phosphatase, 60S ribosomal protein L18a, NRDE2, Acetyl-CoA carboxylase, Propionyl-CoA carboxylase beta chain, actin-related protein 2/3 complex subunit, UPF0565 protein C2orf69, charged multivesicular body protein, alpha-(1,6)-fucosyltransferase, and C-type lectin domain family 2 member D3. A complete list of DMCs between the two *D. pulex* genotypes and their methylation levels are presented in supplementary table S9, Supplementary Material online. In *D. magna*, the differentially methylated HM containing genes are primarily enriched for cell cycle regulation, mRNA processing, and pathways altered by viral infections (fig. 4*E*, supplementary table S10, Supplementary Material online). While the genes containing only LM are marginally enriched for “Nitric oxide stimulates guanylate cyclase.” In *D. pulex* the differentially methylated HM containing genes are enriched for transforming growth factor (TGF) beta signaling and pathways related to translation initiation. The genes containing only LM are enriched for chaperone activity and transcription initiation (fig. 4*E*, supplementary table S10, Supplementary Material online).

### Gene expression and DNA methylation comparison between *D. magna* Strains

We compared gene expression between two *D. magna* strains Bham2 and Xinb3 using age match control samples (*n* = 8). More than half of the genes (13,527/21,293) appear differentially expressed (adjusted *P* value < 0.05, supplementary table S11, Supplementary Material online). This undoubtedly contains a lot of changes that are due to technical differences rather than biological origin, such as differences in sample preparation, library construction, sequencing, mapping and downstream processing. To alleviate some of the technical bias we excluded overlapping genes (7,033) from the analysis. In the reduced set about half of the differentially expressed genes (8,077/14,260; adjusted *P* value < 0.05) have higher expression in Bham2 (4,209/8,077 genes), compared to Xinb3 (3,868/8,077 genes). These genes are enriched for RNA processing (NMD), amino acid synthesis, translation, development and neuronal signaling (SLIT/ROBO) in 14 Reactome pathways. When we analyzed the enrichment separately for genes that have higher expression in one strain, we observed that most of these pathways are coming from genes that have higher expression in Bham2. Additionally we find 85 enriched pathway that are not found in the combined analysis, including many of the same pathways (Defective CFTR causes cystic fibrosis, ABC transporter disorders, Interleukin-1 family signaling, mRNA Splicing, Cyclin A and E associated events) identified in the methylation comparison between the two strains (supplementary table S10, Supplementary Material online). Conversely we only find 3 significantly enriched pathways for the genes that have higher expression in Xinb3, all of which are related to gene expression regulation (supplementary table S11, Supplementary Material online).

Encouraged by the shared pathways found in both DNA methylation and expression analysis, we compared the gene expression and DNA methylation at individual gene level. We selected DMCs that were located within exons 2–4 with methylation changes greater than 50% (907 DMCs in 473 genes). When we compared the direction of methylation changes in the DMCs to the direction of expression changes in the same gene in Bham2 vs. Xinb3, we observed that the direction is the same more often than expected by chance (chi-squared = 7.8617, *P* value = 5.049e−3). Genes where the expression is higher in Bham2 compared to Xinb3 also have higher methylation in the DMCs in Bham2 and vice versa. When we further limited the data to only include genes with statistically significant (adjusted *P* value < 0.05) and a large expression change (log2 fold change > 2; 163 DMCs in 71 genes), the enrichment becomes even stronger (chi-squared = 84.622, *P* value = 3.613e−20), with more than 69% of the genes having the same direction of expression and methylation changes (supplementary table S12, Supplementary Material online).

### Intrinsic and Extrinsic Factors Alter the Methylome of *Daphnia*

**Fig. 5.**
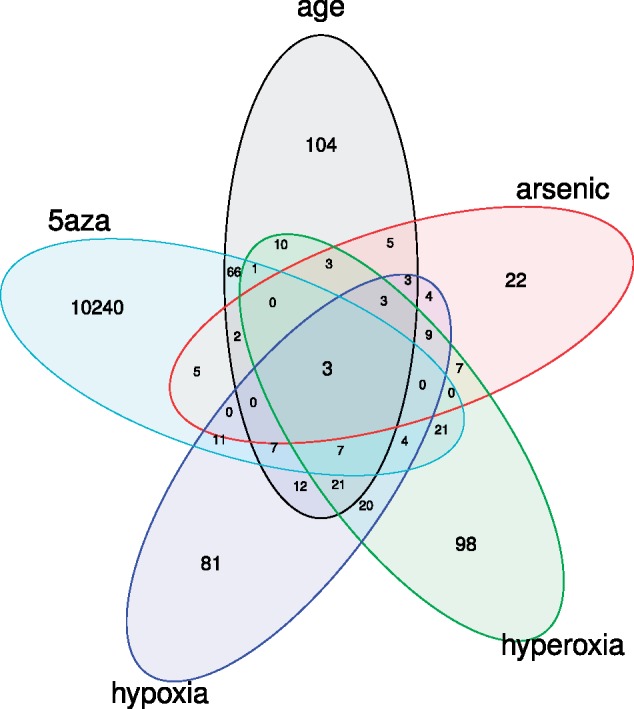
—Venn diagram of differentially methylated CpGs (DMCs) in *D. magna* Bham2. The *Daphnia* were exposed to different stress conditions (arsenic, hypoxia, hyperoxia, 5ʹ-azacitidine) and normal ageing process (5 vs. 14 day old *Daphnia*), showing a relatively small amount of overlap in the DMCs among the conditions.

**Fig. 6.**
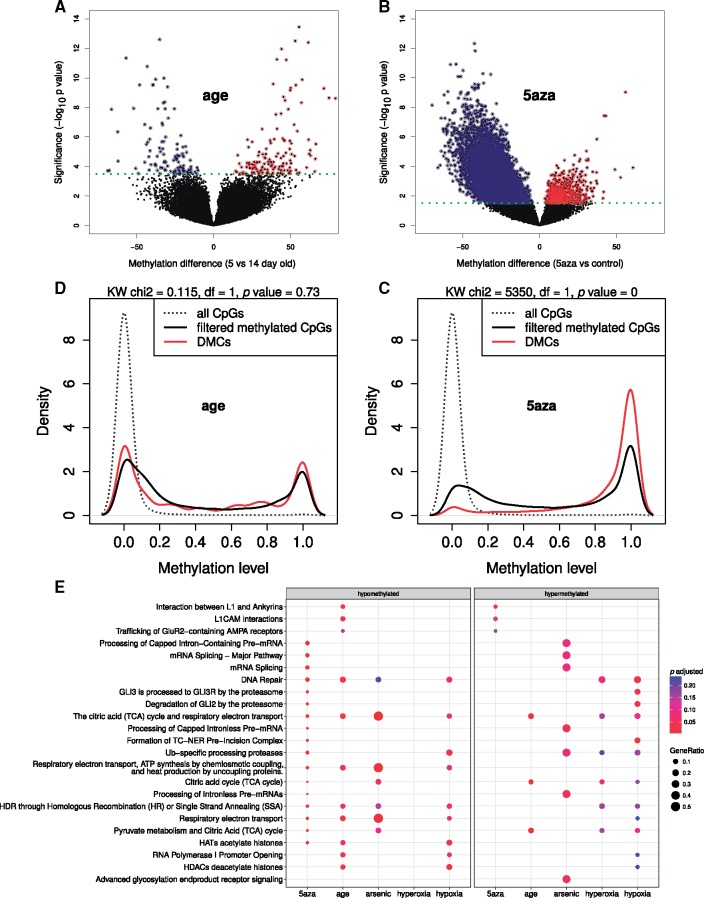
—Extrinsic and intrinsic induced differentially methylated CpGs (DMCs) in *D. magna* Bham2. (*A*) Volcano plot of DMCs in age comparison. Methylation difference is the percentage change in methylation level in 5- to 14-day-old *D. magna* Bham2 (blue = hypomethylated and red = hypermethylated in 5-day-old compared to 14-day-old samples). (*B*) Volcano plot of DMCs in 5aza-treatment. (*C*) Density plot of the DMCs in 5aza-treatment. Majority of the affected CpGs have high methylation level in control samples (red) compared to the unaffected CpGs (black). (*D*) Density plot of the DMCs in ageing (red). The methylation level of affected CpGs is the same as the background set of filtered CpGs (black). Both sets are enriched for higher methylation compared to the unfiltered CpGs (dashed line). (*E*) Enrichment analysis of DMCs across the conditions. The analysis is carried out separately for genes containing hyper- and hypo-methylated CpGs.

DNA methylation acts as an interface between the genome and the environment. Therefore, in order to investigate if DNA methylation in *Daphnia* is sensitive to intrinsic and extrinsic factors, changes in the methylome of *D.magna* Bham2 strain were investigated as a function of age (comparing 5 and 14 day olds) and experimental conditions: arsenic (14 days of exposure at 100 µg L^−1^), hypoxia (continuous low oxygen concentration of 2 mg L^−1^ for 14 days), hyperoxia (continuous oxygen concentration of 8 mg L^−1^ for 14 days) and 5-azacytidine (5 days of exposure at 3.7 mg L^−1^). Interestingly, there is little overlap between the lists of differentially methylated CpGs (DMCs) in the different conditions. The highest overlap was observed between hypoxia and hyperoxia where 36% of the differentially methylated CpGs were shared (fig. 5, supplementary table S13, Supplementary Material online). As shown in figure 5 and supplementary table S13, Supplementary Material online, 5-azacytidine treatment induced the highest number of DMCs. All conditions resulted in an even number of hypo- and hyper-methylated DMCs, as shown in figure 6*A* for age comparison and supplementary table S13, Supplementary Material online, with an exception of 5-azacytidine where 95% of the DMCs are hypomethylated as expected (fig. 6*B* and supplementary table S13, Supplementary Material online). Furthermore, while the DMCs for 5-azacytidine treatment are significantly enriched (Kruskal–Wallis rank sum test chi-squared = 5,350, *P* value < 2.2e−16) for the category of HM (fig. 6*C*), all other exposures are significantly under-represented in HM (supplementary fig. S4, Supplementary Material online). The DMCs in ageing on the other hand represent both HM and LM evenly (fig. 6*D*). As shown in supplementary figure S5, Supplementary Material online, there is a significant enrichment for the genes categorized as common (evolutionarily conserved) in the differentially methylated genes in 5-azacytidine group while other conditions resulted in either marginal or no significant enrichment of a distinct gene category (species specific, *Daphnia*/hymenoptera specific, arthropod/mammal specific or common). The DMCs for the treatment comparisons are enriched for a few shared pathways (fig. 6*E*). For example, 5-azacytidine resulted in a substantial number of pathways being enriched, including mRNA processing, DNA repair, TCA cycle, with majority belonging to the category of hypomethylated CpGs (supplementary table S14, Supplementary Material online).

### Conserved and Emerged Correlation Between DNA Methylation and Gene Expression across Taxa

**Fig. 7.**
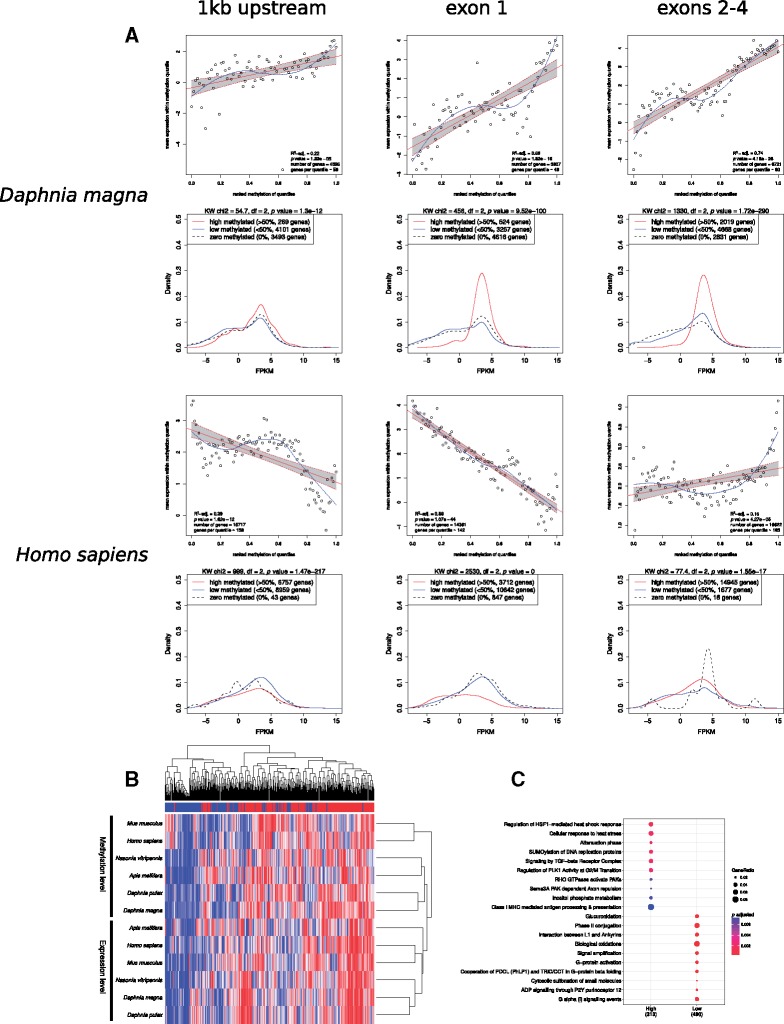
—Correlation analysis of gene expression and DNA methylation data. (*A*) Top panel per species: regression of gene expression and methylation levels across genomic features (1 kb upstream of first exon, exon 1, and exons 2–4). Linear regression and 95% confidence interval marked in red. A loess fit is shown in blue. The legend shows *R*^2^ and *P* value for the linear regression as well as the number of genes used in the analysis and the average number of genes in each quantile. The methylation levels of genes were ranked and the gene expressions were averaged for all genes within the same methylation quantile. Bottom panel per species: density plots of genes relative to their expression level (FPKM). The high methylated genes (red) are over-abundant in the high expression range, in all the features show, compared to LM (blue) and non-methylated (black) genes in arthropods (*D. magna*). Whereas the vertebrates (*H. sapiens*) show over-abundance of high methylated genes only in exons 2–4. In exon 1 and 1 kb upstream from the first exons vertebrates have the opposite pattern; over-abundance of LM genes in the high expression range. Differences in the expression densities among methylation states are analyzed with Kruskal–Wallis sum rank test. (*B*) Heatmap of rank ordered mean methylation and mean expression level of genes belonging to the same cluster of orthologous genes (414 orthogroup). The methylation level was calculated from exons 2–4 in arthropods and exon 1 in vertebrates. In vertebrates, the rank order was reversed as methylation correlates negatively with gene expression (in exon 1). (*C*) Enrichment analysis of the two main clusters in the heatmap. The “High” cluster has high expression in most species and high methylation in arthropods (exons 2–4) and low methylation in vertebrates (exon 1). The “Low” cluster has low expression across species and low methylation in arthropods (exons 2–4) and high methylation in vertebrates (exon 1).

The correlation between methylation status and gene expression level was investigated across species using matching RNA-seq data sets generated for *D. pulex* EB45 (GSE103939) and supplemented with publicly available data sets from GEO, the ENCODE project, and the *D.magna* transcriptome study (Orsini et al. 2016) (supplementary table S1, Supplementary Material online). Genes were categorized by their methylation level [methylation level above (HM) and below (LM) 50%] for genomic features exon 1, exons 2–4, and 1 kb upstream from the first exon. Interestingly, as shown in figure 7*A* in both vertebrates and invertebrates the genes containing HM in exons 2–4 were enriched for high expression. Genes containing HM in 1 kb upstream and exon 1 were also enriched for high expression in invertebrates, whereas in vertebrates the LM were enriched for these features (see fig. 7*A* and supplementary fig. S6, Supplementary Material online for statistical analysis).

Genes were grouped by their methylation level into ranked quantiles for genomic features 1 kb upstream, exon 1, exons 2–4, and the expression level was averaged for those genes. As shown in figure 7*A* and supplementary figure S6, Supplementary Material online, for all species there is a statistically significant positive correlation between methylation level for exons 2–4 and gene expression level, although as expected the linear correlation was much more pronounced for invertebrates than vertebrates (*P* values ranging from 4.18e−26 to 4.27e−05). However, the difference between invertebrates and vertebrates emerges when the methylation level for exon 1 and 1 kb upstream regions were regressed against gene expression level. While in invertebrates there is a significant positive correlation (see supplementary fig. S6, Supplementary Material online), in vertebrates there is a statistically significant negative correlation between average methylation levels in exon 1 (e.g. adjusted *R*^2^ and *P* value for *H. sapiens* are: 0.86 and 1.07e−44) and 1 kb upstream (less pronounced) and gene expression level (e.g. adjusted *R*^2^ and *P* value for *H. sapiens* are: 0.39 and 1.62e−12; fig. 7*A*).

Furthermore, we combined the DNA methylation and gene expression data for all species. This was achieved by first identifying conserved orthologous gene groups in each species, where we had sufficient data on both expression and methylation levels (414 orthogroups, supplementary table S15) and then calculated the mean expression and methylation levels for these orthogroups. For the arthropod species, methylation levels were based on exons 2–4 and for vertebrates based on exon 1, as these categories strongly correlated with gene expression. As the methylation level was negatively correlated in the vertebrates the scale was reversed for these species. Hierarchical clustering was used to organize the orthogroups based on the mean methylation and expression levels (fig. 7*B*). The orthogroups clustered into two distinct groups: one with “low” methylation (high methylation in vertebrates) and low gene expression levels and another with “high” methylation (low in vertebrates) and high gene expression levels. The genes within these orthogroups are enriched for a variety of pathways deemed essential for survival including stress response, immune system and intracellular signaling (fig. 7*C*, supplementary table S16, Supplementary Material online).

**Fig. 8.**
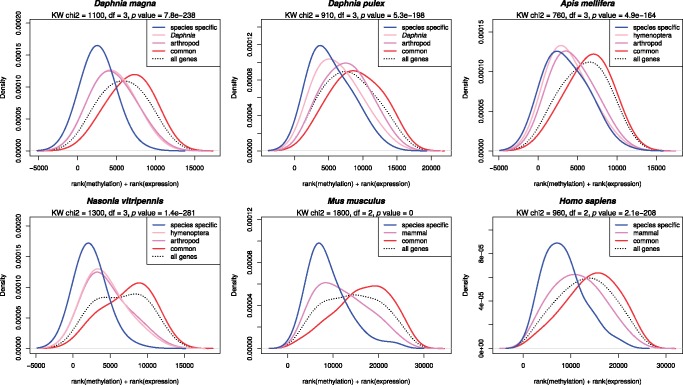
—Evolutionary conservation of methylation and gene expression. Density plot for the sum of ranked order of DNA methylation and gene expression for genes at different levels of evolutionary conservation. The average methylation level of genes at exons 2–4 were ranked and combined with the rank order based on gene expression level. Genes were separated into six distinct categories based on the evolutionary conservation level identified with OrthoFinder (species specific, *Daphnia*/hymenoptera specific, arthropod/mammal specific, and common). *x* axis from right to left: highest methylation/expression to lowest methylation/expression.

In addition, to investigate if the genes with high methylation level and high gene expression level tend to be enriched for evolutionarily conserved genes, we calculated the sum of ranked order for methylation and gene expression levels. The genes in our list were separated into six distinct categories (species specific, *Daphnia*/hymenoptera specific, arthropod/mammal specific and common), based on orthologous gene IDs (fig. 8). As shown in figure 8, the genes with high levels of DNA methylation for exons 2–4 and high expression levels were significantly enriched for the common category (evolutionarily conserved), while species specific genes had much lower ranked value for DNA methylation level and gene expression (fig. 8). As shown in figure 8, there is a decrease in the density of common > arthropod > *Daphnia* specific and > species specific genes moving from high to low ranked sum of DNA methylation level and gene expression level (*x* axis). This analysis showed that the distribution of species specific, *Daphnia*/hymenoptera specific, arthropod/mammal specific or common genes are statistically significantly different based on the sum of ranked values for DNA methylation and average expression level.

## Discussion

In this study, we were interested in understanding the role of DNA methylation in *Daphnia* species and compared it to selected vertebrate and invertebrate species. We aimed to understand how DNA methylation levels across genomic features correlates with gene expression and to achieve a better understanding of the potential function of DNA methylation in *Daphnia*. We showed that strain specific differences in DNA methylation co-vary with gene expression differences. Finally, we identified a set of methylated evolutionary conserved genes in *Daphnia* which are potentially regulated in the same manner.

To understand the role of DNA methylation variations in *Daphnia* species, we performed whole genome bisulfite sequencing (WGBSeq) on two *Daphnia* species (*D. magna*, Bham2 and *D. pulex* EB31 and EB45) in the context of mild stress treatments and the natural process of ageing. As expected, the treatments resulted in moderate changes in CpG methylation. The altered CpGs were mainly unique for each treatment condition. The differentially methylated genes were enriched for pathways primarily related to cell to cell signaling; G-protein coupled receptor (GPCR) signaling, IP3 and IP4 synthesis, and ion and small molecule transport (fig. 4*A*). Stress-induced changes in DNA methylation have been shown to cause long-term physiological effects in model organisms that are mediated by alterations in gene expression (Murgatroyd et al. 2009; Dowen et al. 2012; Wu et al. 2014). Furthermore, we exposed the *Daphnia* to 5-azacytidine-treatment (5aza), a potent methylation inhibitor (Christman et al. 2002). This treatment severely reduced the methylation levels of CpG sites, especially at the high methylated CpGs (HM, fig. 6*B*). All the other treatments except for 5aza (and ageing) were significantly under-represented in the HM changes. This result indicates that the HM are actively and continuously maintained at high methylation levels via DNMT. It is known that 5aza is capable of inhibiting a wide range of critical cellular functions, such as RNA, DNA, and protein synthesis as demonstrated by us and others (fig. 4*E*) (Christman et al. 1983; Creusot et al. 1982). This suggests that the HM in *Daphnia* could also be crucial for cellular integrity and functions. Not surprisingly the genes altered by 5aza were mainly enriched for evolutionary conserved genes. This was not surprising as we have shown that HM mainly occur in conserved genes while LM mainly occur in species specific genes (fig. 2*A*).

Interestingly the methylation profile at the gene level is very similar between the two *Daphnia* species. *Daphniamagna* and *D. pulex* are among the most distantly related species within the genus *Daphnia* with previous estimates on the basis of a mitochondrial molecular clock suggested a divergence time of 200 MY (Haag et al. 2009). Yet more than 70% of the genes analyzed have similar methylation levels (29% high and 43% low methylation) in both species. The genes that have substantially different methylation levels between the species are enriched for entirely different pathways. The genes with high methylation in *D. magna* and low methylation *D. pulex* are enriched for DNA damage recognition and repair, while genes with higher methylation in *D. pulex* are enriched for extracellular matrix organization and cell to cell signaling.

The overall methylation pattern across the genomic features is the same between the different genotypes and strains of *Daphnia*. However, there are differences in global and gene specific methylation levels between the investigated *Daphnia* strains and genotypes, particularly between the two *D. magna* strains. It has been reported that genetic differences between *Daphnia* populations can be quite strong (Haag et al. 2009). This is evident as we look at the amount of SNP variation between the two distantly related *D.magna* strains and the two closely related *D. pulex* genotypes. The level of genetic diversity between the two *D. magna* strains in terms of fixed SNPs is about five times greater compared to the number of fixed SNPs between *D. pulex* genotypes. This corresponds with a higher level of difference observed in both global and gene specific DNA methylation between the two *D. magna* strains compared to *D. pulex* genotypes. The differences in global methylation levels are 16 times greater between *D. magna* strains (1.51% vs. 1.03%) compared to *D. pulex* genotypes (0.44% vs. 0.41%). And the number of DMCs is 14 times greater between *D. magna* strains compared to *D. pulex* genotypes (20,656 vs. 1,442 DMCs).

The differentially methylated genes were divided into two categories of exclusively containing LM CpGs and exclusively containing high methylated CpGs (HM). Enrichment analysis showed that the HM containing differentially methylated genes were enriched for non-overlapping pathways in *D. magna* and *D. pulex*. For example, *D. magna* strains differed in methylation of pathways related to cell cycle regulator, RNA processing and viral infection while methylation differences between *D. pulex* genotypes related to genes associated with pathways, such as TGF-signaling and RNA translation (fig. 4*E*). Most interestingly, the majority of the differentially methylated genes between the different strains and genotypes were detected at the LM CpG sites and these genes were not overly enriched for specific pathways (fig. 4*C*–*E*).

We observed a correspondingly large number of gene expression differences in non-overlapping genes (8,077/14,260 genes, with adjusted *P* value < 0.05) between the two *D. magna* strains Bham2 and Xinb3. The gene expression analysis between the two strains identified similar enriched pathways as the methylation analysis (in particular mRNA splicing and cell cycle regulators). Also the DMCs with a large methylation change significantly co-varied with gene expression, when the DMCs were located in exons 2–4. This covariation was strengthened when we limited the data to include only genes with large expression changes.

Similar patterns in methylation variation have been observed by others when comparing different species of *Daphnia* (Asselman et al. 2016). As reported by Asselman et al. (2016) genes with variable methylation levels between species tend to be also responsive in gene expression changes when subjected to experimental manipulations. However, the majority of the genes that show plastic and adaptive variations tend to have exceptionally low levels of methylation (<5%) while the high methylated genes in *Daphnia* show almost no variation between species, and appear to be more conserved (Asselman et al. 2016).

In order to extend our findings beyond *Daphnia* and to put them in a larger evolutionary context, we compared the DNA methylation profile of *Daphnia* to other vertebrate and arthropod species. Although the methylation profiling for our chosen species were not conducted on matching tissue types (see supplementary table S1, Supplementary Material online for sample source descriptions), this limitation did not impact the higher order analysis and interpretation of methylation pattern across our species. In invertebrate species, DNA methylation is sparse and occurs mostly in gene bodies (Wang et al. 2013; Keller et al. 2016; Glastad et al. 2017). In *Daphnia*, the methylation landscape exhibits a flat, near zero, methylation across introns and intergenic regions. The methylation level sharply increases starting from the first exon and reaches maximum levels at exons 2 and 3, and declines starting from exon 4, reaching global background levels near the last exons. The flanking regions (defined as 1 kb up- and down-stream) exhibit higher methylation compared to the global background and have their minimum methylation levels at the start and end of the gene. This pattern was observed in all four arthropod species, with *Daphnia* species demonstrating the lowest level of DNA methylation compared to *N.vitripennis* and *A.mellifera* (fig. 1*B* and *C*, supplementary table S4, Supplementary Material online). Furthermore, *Daphnia* species, similar to all other species investigated in this study, display a full repertoire of DNMTs (fig. 1*A*, supplementary fig. S2, Supplementary Material online). In contrast to arthropods, DNA methylation in vertebrates is ubiquitous and occurs at relatively high levels and is often near saturation level within gene bodies. In vertebrates, the upstream region and first exon appear to be suppressed for methylation, particularly at the very start of the gene, and the level of methylation sharply increases after the first exon to global background levels. Although the levels of DNA methylation and the distribution of methylated CpG sites across the genome differ between vertebrates and invertebrates, there are significant similarities. For example, the methylation percentage across the genome, promoter region and gene body, follows a characteristic bimodal distribution for both vertebrates and invertebrates, indicating that this may be an evolutionarily conserved pattern (Keller et al. 2016). Most interestingly, our data, similar to previous findings (Asselman et al. 2016), supports the idea that the high methylated genes are more evolutionarily conserved and enriched for basal cellular functions, while LM genes are mainly enriched for species specific genes (fig. 2*A*).

DNA methylation in vertebrates has been typically associated with transcriptional repression and suppression of transposable elements (Jones and Takai 2001; Gibbs et al. 2010; Bell et al. 2011). However, it is becoming increasingly apparent that the function of DNA methylation is context and location dependent. In vertebrates, methylation at the promoter regions and first exons has been shown to correlate negatively with gene expression (Brenet et al. 2011), while methylation within the rest of the gene body has a significant positive correlation with gene expression (Lev Maor et al. 2015; Li et al. 2017). In arthropods, this negative relationship between gene expression and DNA methylation (fig. 7*A*, supplementary fig. S6, Supplementary Material online) at the first exon and the promoter region (1 kb upstream of the first exon) does not hold. Instead, methylation in arthropods has either a positive or weak correlation with gene expression at the gene body and 1 kb upstream region, respectively (fig. 7*A*). Similar to vertebrates (Li et al. 2017), the positive correlation between gene expression and DNA methylation in the internal exons, specifically exons 2–4, is particularly strong in invertebrates (fig. 7*A*, supplementary fig. S6, Supplementary Material online). In addition, our data demonstrate that pathways associated with genes with high methylation levels in invertebrates (low methylation level at first exon in vertebrates) and high expression levels in both vertebrates and invertebrates are evolutionarily highly conserved and enriched for the same pathways across the invertebrate–vertebrates boundary (figs. 7*C* and 8). While the less conserved and faster evolved genes tend to have low methylation levels and are potentially contributing towards adaptation and strains specific differences.

In vertebrates majority of the internal exons are heavily methylated (Li et al. 2017). Therefore, it is possible that the negative impact of DNA methylation at promoter regions, and first exons has been evolved as a secondary mechanism to prevent high levels of expression from heavily methylated genes (Tirado-Magallanes et al. 2017). Interestingly, in vertebrates not only are all internal exons heavily methylated, but also introns have a higher level of methylation compared to invertebrates. One possible explanation for this difference could be linked to an increase in both intron length, average number of spliced isoforms per gene, and differences in splicing regulation that has emerged in vertebrates (Gelfman et al. 2012). In vertebrates, the size of many introns has grown to thousands of nucleotides while the tight selection on exon length has been evolutionarily maintained (Lev Maor et al. 2015). Along with this increase in intron length the methylation levels in introns has dramatically increased. Thus, it is possible that the changes observed in the gene body methylation level of vertebrates could be linked to regulation of splicing and exon skipping (Kim et al. 2007).

In conclusion, we hypothesize that the negative effect of DNA methylation on gene expression is a novel mechanism that evolved in the vertebrate lineage, to counterbalance increased global methylation levels. Emergence of this novel regulatory role for DNA methylation can be observed in the early chordate, *Ciona intestinalis* (Keller et al. 2016). Subset of promoters in *C.intestinalis* demonstrate low levels of methylation and are correlated with high levels of gene expression. However, *C.intestinalis* still retains its ancestral high methylated promoters that correlate positively with gene expression (Keller et al. 2016). Irrespective of the potential new function of DNA methylation in the vertebrate lineage, we show that the positive correlation between gene expression level and DNA methylation level is evolutionary conserved.

## Supplementary Material

Supplementary data are available at *Genome Biology and Evolution* online.

## Supplementary Material

Supplementary Data
