# Supplementary material for: Pattern of DNA Methylation in Daphnia: Evolutionary Perspective
Source: Genome Biol Evol. 2018 Jul 30;10(8):1988–2007. doi: 10.1093/gbe/evy155 (PMC6097596; doi:10.1093/gbe/evy155)
Supplement: Supplementary Data [file evy155_kvist_et_al_supplements.zip › S4_Fig_Methylation profile of DMC in exposures.pdf]

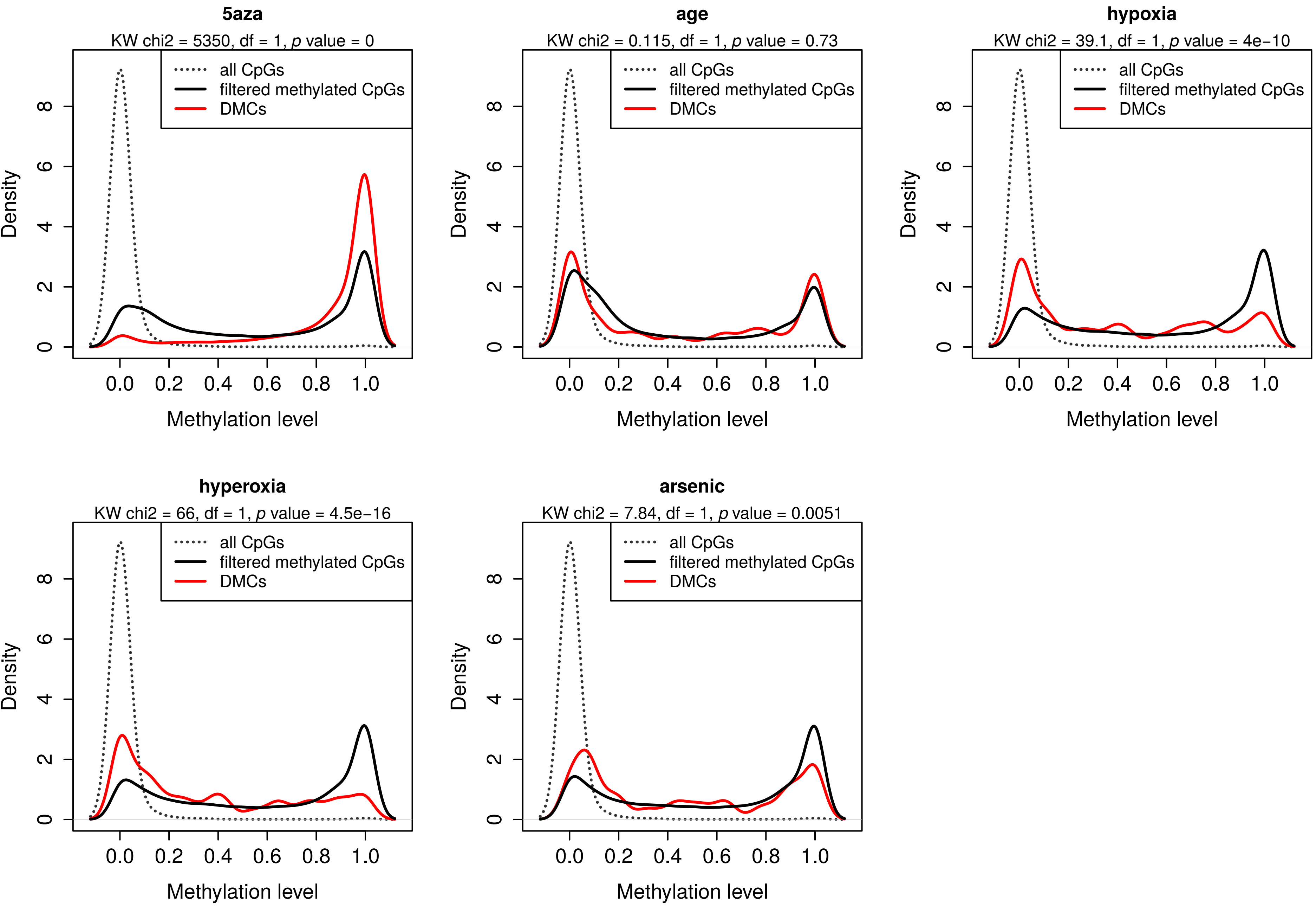
5aza
KW chi2 = 5350, df = 1, p value = 0
age
KW chi2 = 0.115, df = 1, p value = 0.73
hypoxia
KW chi2 = 39.1, df = 1, p value = 4e−10
hyperoxia
KW chi2 = 66, df = 1, p value = 4.5e−16
arsenic
KW chi2 = 7.84, df = 1, p value = 0.0051
all CpGs
filtered methylated CpGs
DMCs
Density
Methylation level
0
2
4
6
8
0.0
0.2
0.4
0.6
0.8
1.0
