# Supplementary figures and images for: Pattern of DNA Methylation in Daphnia: Evolutionary Perspective
Source: Genome Biol Evol. 2018 Jul 30;10(8):1988–2007. doi: 10.1093/gbe/evy155 (PMC6097596; doi:10.1093/gbe/evy155)

Median centered change in coverage (log10 coverage - log10 median)

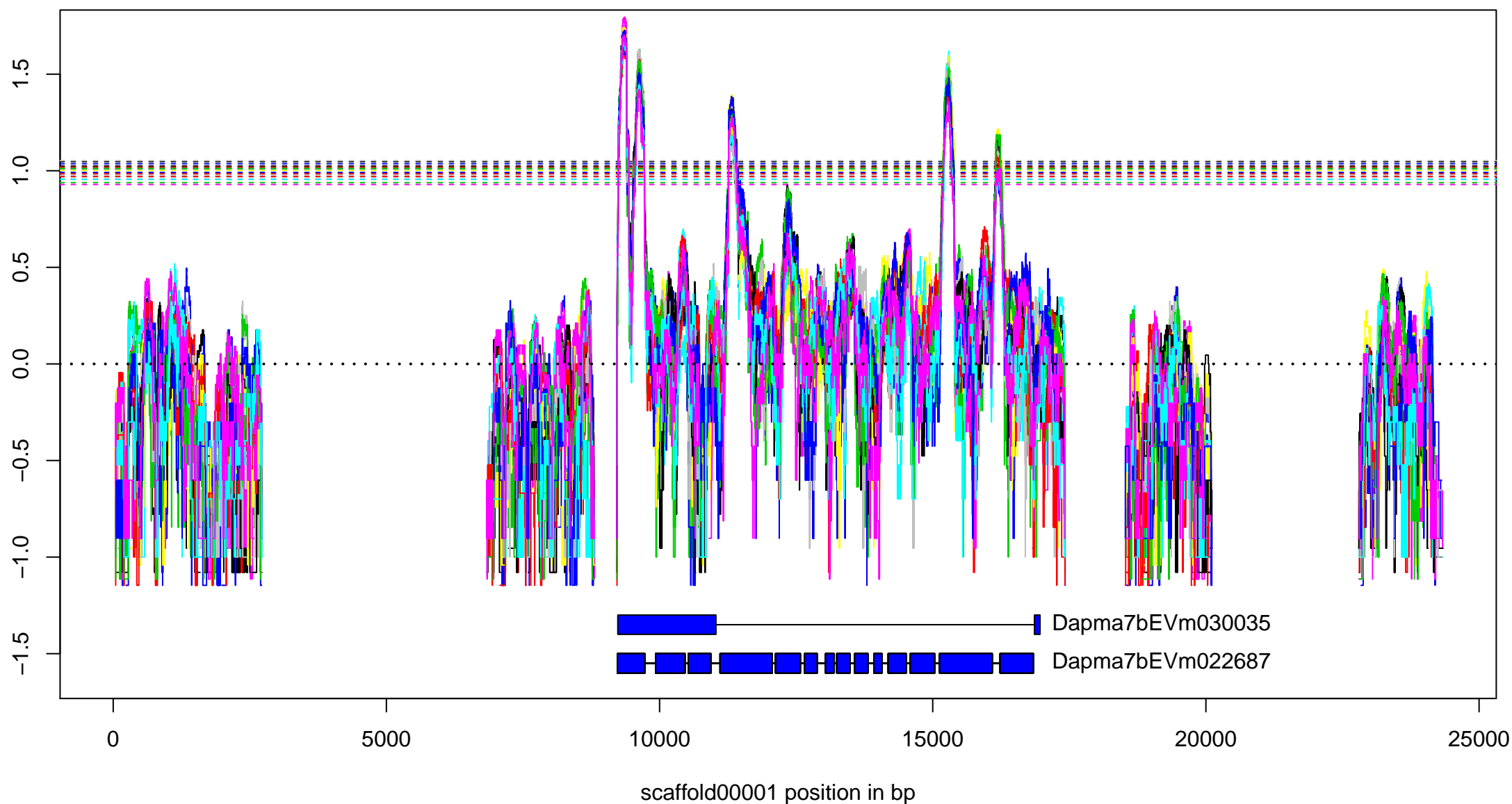

Supplement: Supplementary Data [file evy155_kvist_et_al_supplements.zip › S1_Fig_Excessive coverage in scaffold00001.pdf]

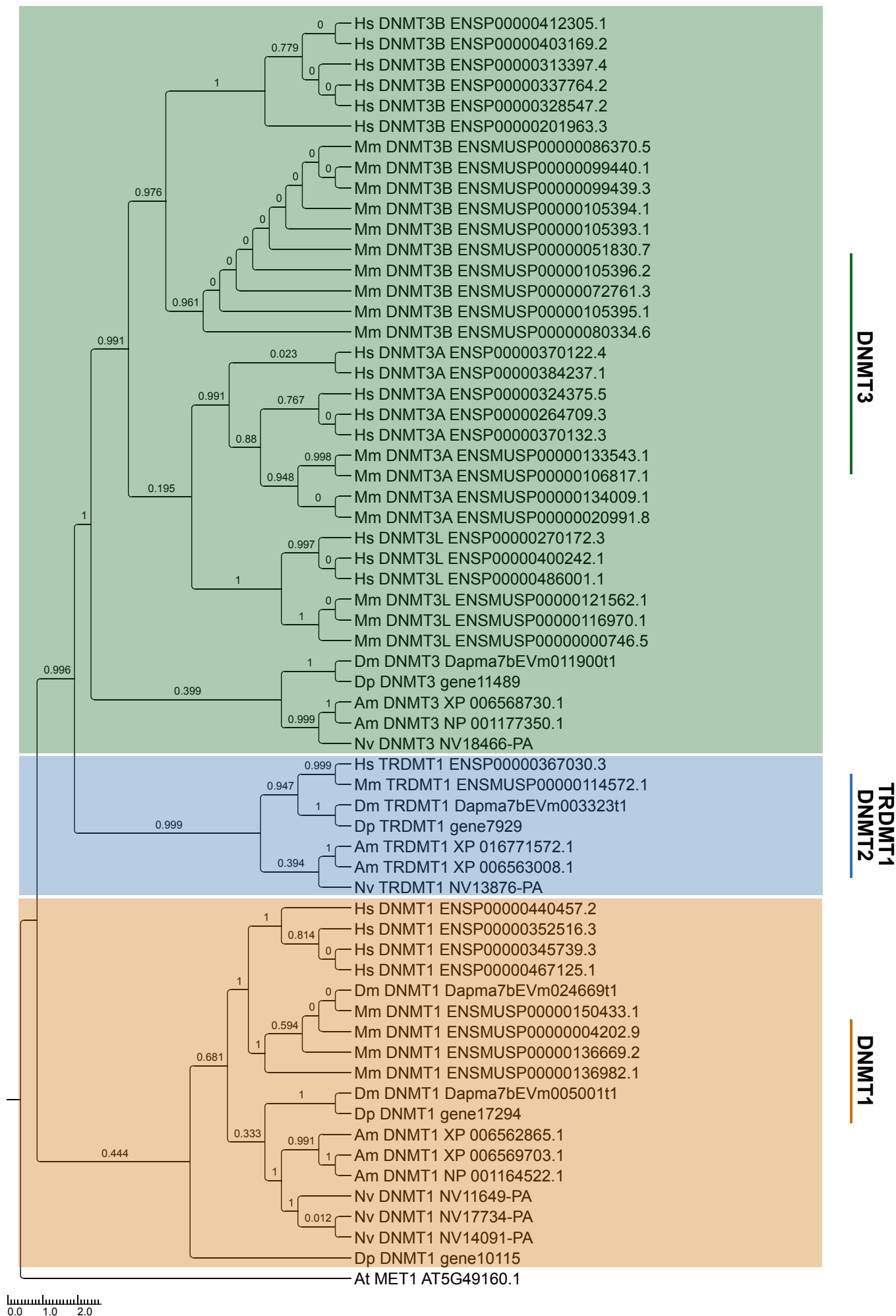

Supplement: Supplementary Data [file evy155_kvist_et_al_supplements.zip › S2_Fig_PhyML_DNMT_genes.pdf]

**A*****Daphnia magna* Bham2 vs Xinb3**Xsq= 8.6, *p* value= 0.034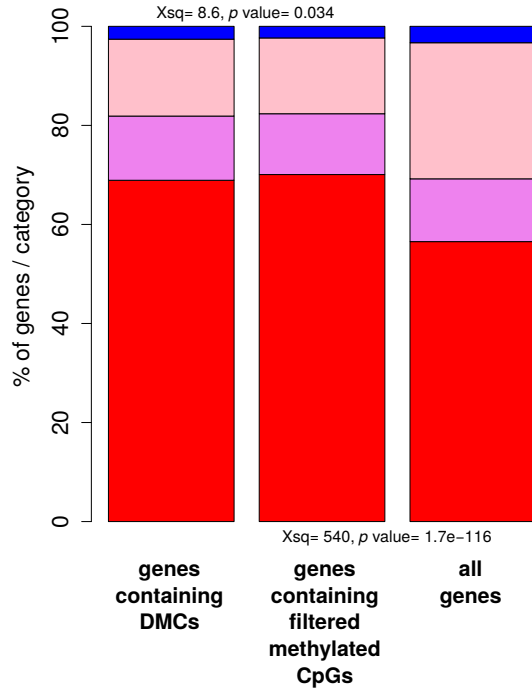**B*****Daphnia pulex* EB31 vs EB45**Xsq= 18, *p* value= 5e-04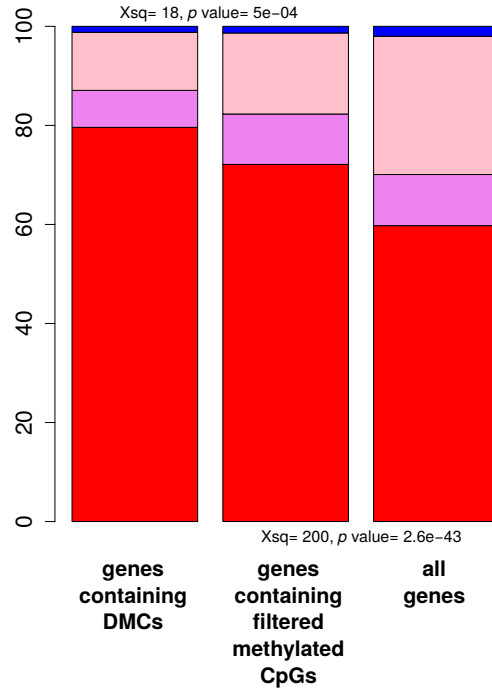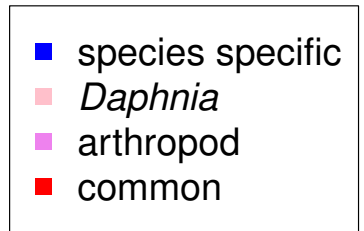

Supplement: Supplementary Data [file evy155_kvist_et_al_supplements.zip › S3_Fig_Evolutionary conservation of strain variable genes.pdf]

### 5aza

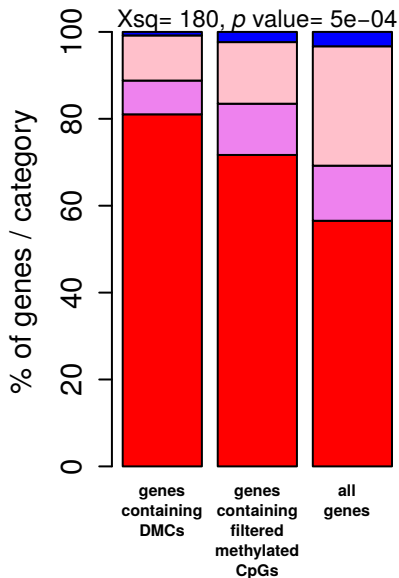

### age

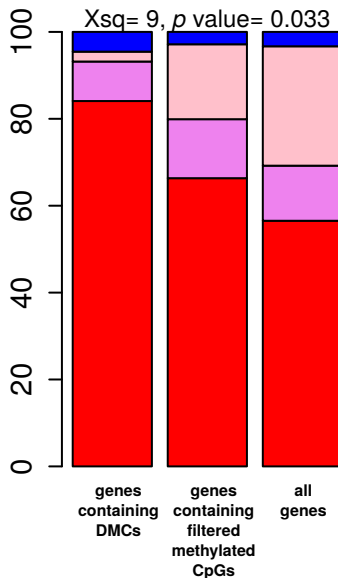

### hypoxia

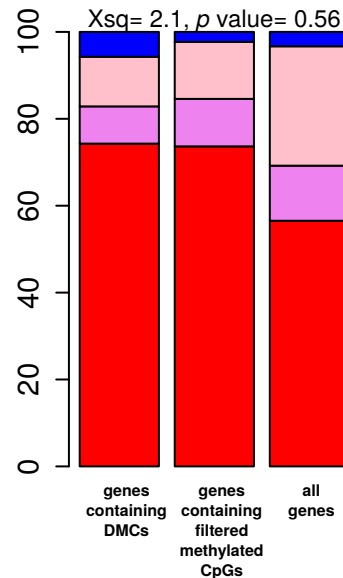

### hyperoxia

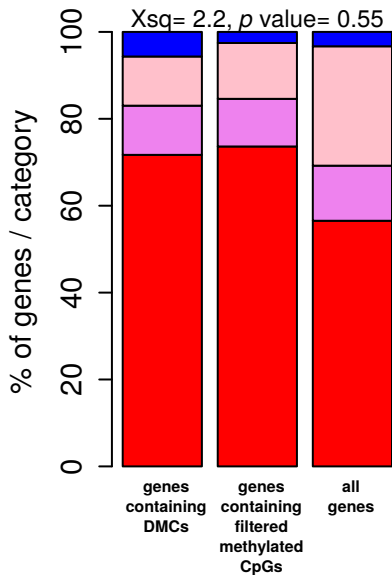

### arsenic

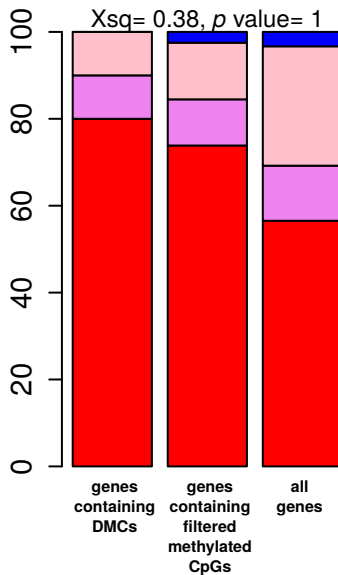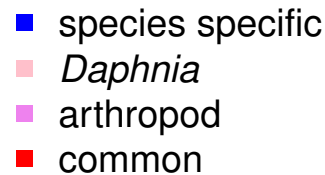

Supplement: Supplementary Data [file evy155_kvist_et_al_supplements.zip › S5_Fig_Evolutionary conservation of exposure DMCs.pdf]

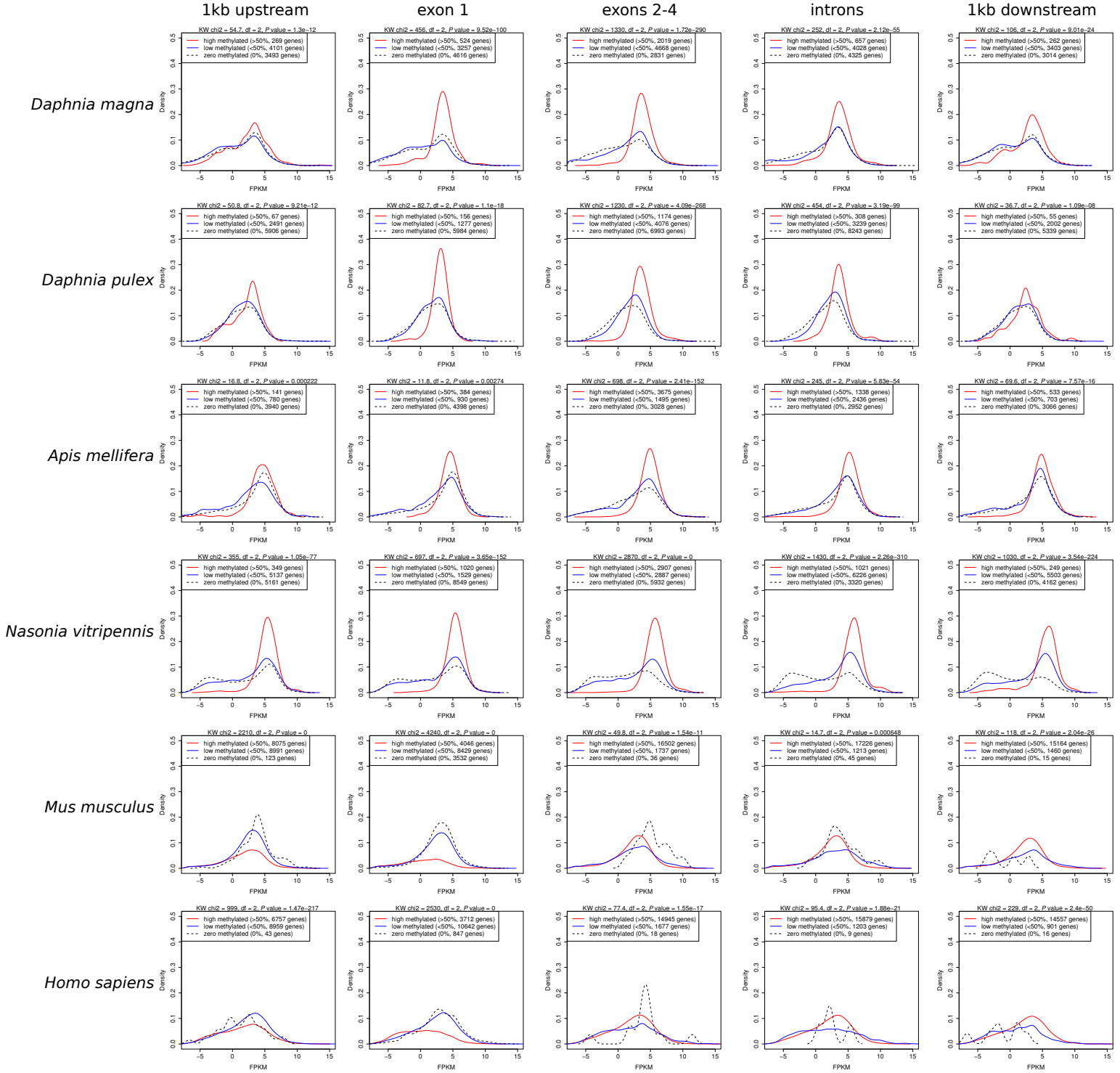

*Daphnia magna*

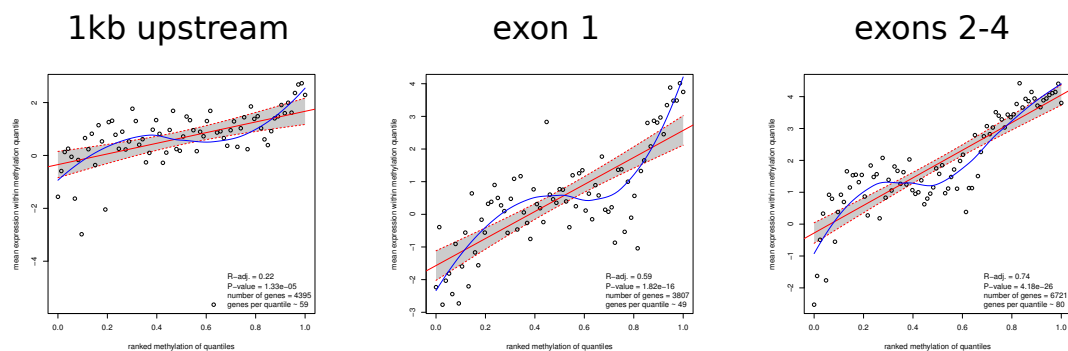

*Daphnia pulex*

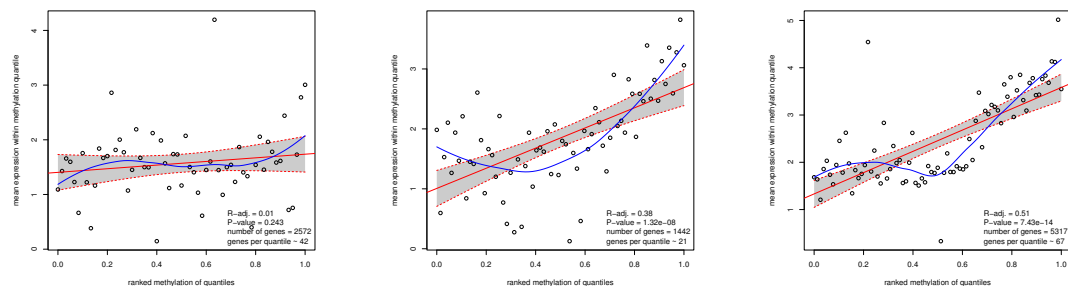

*Apis mellifera*

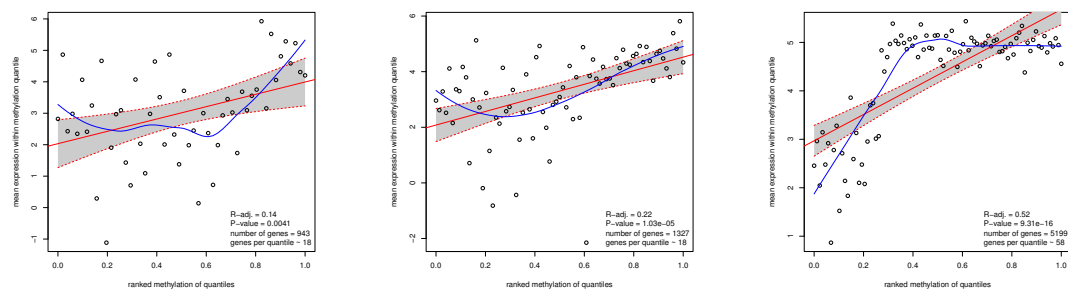

*Nasonia vitripennis*

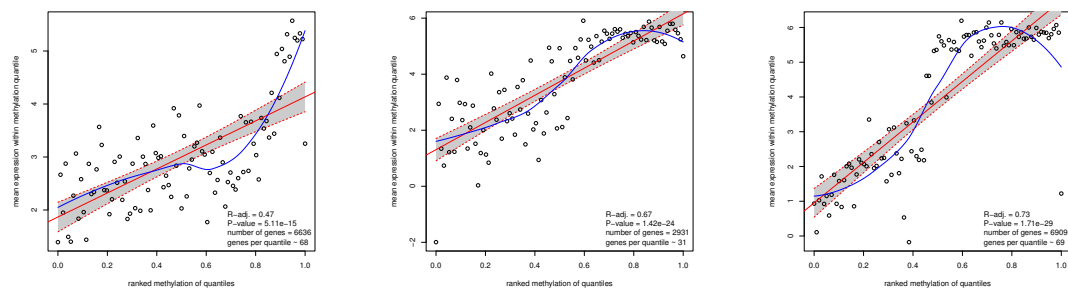

*Mus musculus*

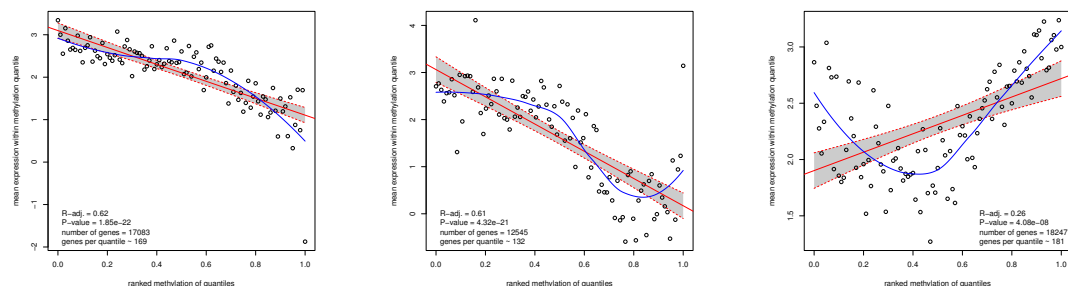

*Homo sapiens*

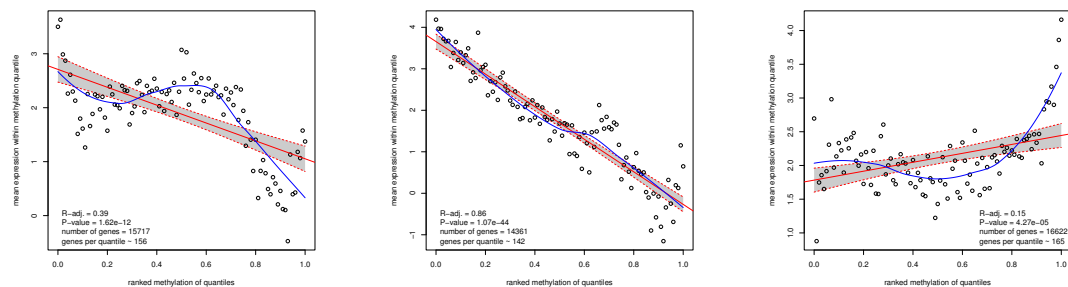

Supplement: Supplementary Data [file evy155_kvist_et_al_supplements.zip › S6_Fig_Expression densities and regressions by methylation.pdf]
